# Programmed Cell Death in Stigmatic Papilla Cells Is Associated With Senescence-Induced Self-Incompatibility Breakdown in Chinese Cabbage and Radish

**DOI:** 10.3389/fpls.2020.586901

**Published:** 2020-12-07

**Authors:** Jiabao Huang, Shiqi Su, Huamin Dai, Chen Liu, Xiaochun Wei, Yanyan Zhao, Zhiyong Wang, Xiaowei Zhang, Yuxiang Yuan, Xiaolin Yu, Changwei Zhang, Ying Li, Weiqing Zeng, Hen-Ming Wu, Alice Y. Cheung, Shufen Wang, Qiaohong Duan

**Affiliations:** ^1^State Key Laboratory of Crop Biology, College of Horticulture Science and Engineering, Shandong Agricultural University, Tai’an, China; ^2^Institute of Vegetables and Flowers, Shandong Academy of Agricultural Sciences, Jinan, China; ^3^Institute of Horticulture, Henan Academy of Agricultural Sciences, Zhengzhou, China; ^4^Institute of Vegetable Science, Zhejiang University, Hangzhou, China; ^5^Department of Horticulture, Nanjing Agricultural University, Nanjing, China; ^6^Trait Discovery, Corteva Agriscience, Johnston, IA, United States; ^7^Department of Biochemistry and Molecular Biology, University of Massachusetts Amherst, Amherst, MA, United States

**Keywords:** self-incompatibility, flower senescence, plant senescence, PCD, ethylene, *Brassicaceae*

## Abstract

Self-incompatibility (SI) is a genetic mechanism flowering plants adopted to reject self-pollen and promote outcrossing. In the Brassicaceae family plants, the stigma tissue plays a key role in self-pollen recognition and rejection. We reported earlier in Chinese cabbage (*Brassica rapa*) that stigma tissue showed upregulated ethylene responses and programmed cell death (PCD) upon compatible pollination, but not in SI responses. Here, we show that SI is significantly compromised or completely lost in senescent flowers and young flowers of senescent plants. Senescence upregulates senescence-associated genes in *B. rapa*. Suppressing their expression in young stigmas by antisense oligodeoxyribonucleotide abolishes compatible pollination-triggered PCD and inhibits the growth of compatible pollen tubes. Furthermore, ethylene biosynthesis genes and response genes are upregulated in senescent stigmas, and increasing the level of ethylene or inhibiting its response increases or decreases the expression of senescence-associated genes, respectively. Our results show that senescence causes PCD in stigmatic papilla cells and is associated with the breakdown of SI in Chinese cabbage and in radish.

## Introduction

Self-incompatibility (SI) is a genetic mechanism widely adopted by flowering plants and present in roughly half of the species to promote outcrossing and prevent inbreeding (de Nettancourt, 1977). A prevalent system is the sporophytic SI system in the *Brassicaceae* vegetable plants, such as *Brassica rapa*, *Brassica oleracea*, and *Raphanus sativus* (Nasrallah, 2019; Jany et al., 2019). As the *Brassicaceae* have dry stigmas, only compatible pollen grains are capable of inducing the stigma to release necessary resources, such as water and other factors, but the absence of surface secretions prevents SI pollen or unrelated foreign pollen from adhering, hydrating, and germinating at the earliest stages of pollination (Samuel et al., 2009; Jany et al., 2019). *Brassicaceae* SI is controlled by two genetically linked polymorphic S loci: the pollen-coat-expressed S-locus cysteine-rich protein (SP11/SCR) and the stigma-specific S-locus Ser/Thr receptor kinase (SRK) (Nasrallah, 2019). The recognition of self-pollen is dependent on the interaction of SRK with SP11/SCR of the same S haplotype (Takayama et al., 2001; Kachroo et al., 2001; Ma et al., 2016). Subsequent intracellular signaling pathways in the papilla cells ultimately lead to the rejection of self-pollen either by blocking hydration and germination of pollen or by restricting pollen tube growth into the stigma (Samuel et al., 2009; Indriolo et al., 2012; Sankaranarayanan et al., 2015, 2017; Scandola and Samuel, 2019; Jany et al., 2019).

Self-incompatibility is used as a breeding tool for F1 hybrid production in a broad range of the *Brassicaceae* crops (Nou et al., 1993), as hybrid vigor is highly valued in the agricultural industry. For hybrid seed production, ideal SI inbred lines are required to have strong and stable SI to prevent self-fertilized seeds. For the propagation of SI inbred lines, SI is required to be broken down in order to form self-fertilized seeds. However, the underlying mechanism regulating SI strength remains largely unknown. It has been reported that transforming the cognate pair of SCR-SRK into Arabidopsis ecotype Col-0 transits the self-compatible (SC) *Arabidopsis thaliana* into SI, but senescent flowers, i.e., at late stage 14, in these transformed plants have considerably weakened SI (Nasrallah et al., 2002, 2004; Tantikanjana and Nasrallah, 2012). In wild radish and in other species, older unpollinated flowers or flowers from older plants discriminate less between outcrossed pollen and self-pollen and are more prone to self-fertilize (Marshall et al., 2010). In the breeding industry, these old unpollinated flowers and flowers from old plants would allow the production of self-fertilized seeds, thus compromising the purity of desired hybrid seeds. Therefore, it is important to understand the underlying mechanism regulating senescence-associated breakdown of SI, which has not been characterized at all.

Programmed cell death (PCD) is known to be important and highly regulated during plant growth and development, successful sexual reproduction, and response to biotic and abiotic environmental stresses (Wu and Cheung, 2000; Xie et al., 2014; Gong et al., 2015; Daneva et al., 2016; Huysmans et al., 2017; Noman et al., 2020; Su et al., 2020; Zhang et al., 2020). For example, in the senescence of leaf or flower in *A. thaliana*, senescence-associated genes (*SAGs*) are highly upregulated (Shahri and Tahir, 2014). Upstream regulator involves the key transcription factors triggering the senescence process, such as ORESARA1 (ORE1) (Wagstaff et al., 2009), which functions as part of a regulatory network downstream of ethylene signaling (Kim et al., 2009, 2014). In the stigma, ORE1-mediated PCD in the papilla cells terminate floral receptivity in aging unpollinated flowers (Gao et al., 2018). Based on transcriptional analysis of compatible and incompatible pollinations in *Brassica napus*, it has been suggested that a rapid stigma senescence response followed compatible pollination (Sankaranarayanan et al., 2013). However, whether the stigma senescence plays a role in the compatible pollination or has merely resulted from compatible pollination, which is common in many angiosperms (Weber and Goodwillie, 2007; Spigler, 2017), was not clear at that time. We showed earlier in *B. rapa* that PCD in the stigmatic papilla cells is triggered by cross-pollination and suppressing PCD inhibits compatible pollination, resulting in a process that resembles the SI responses (Su et al., 2020). We also showed that treating stigmas with ethephon, an ethylene-releasing reagent, induces PCD in the stigmatic papilla cells and breaks down SI in heading Chinese cabbage (Su et al., 2020). These results suggested a close relationship between PCD in the stigmatic papilla cells and pollen germination and penetration of the stigma.

In this study, we utilized Chinese cabbage (*B. rapa* L. spp. *pekinensis*) and radish (*R. sativus*) to investigate the molecular mechanism regulating the strength of SI in senescent flowers and flowers of senescent plants. We focused on the stigmatic papilla cells as the earliest interface for pollen germination and penetration of compatible pollen, or rejection of SI pollen. We show that SI was significantly compromised or completely lost in senescent flowers and young flowers of senescent plants, which is closely associated with senescence-induced PCD in papilla cells. Expression of *BrSAG29* and *BrSAG12* and the transcription factor *BrORE1* were upregulated, suppressing them by antisense oligodeoxyribonucleotide (AS-ODN) in young flowers abrogated compatible pollination-triggered PCD and consequently inhibited the growth and penetration of compatible pollen. Furthermore, ethylene biosynthesis genes and ethylene response genes were upregulated in senescent stigmas, and manipulating the level of ethylene or its response affected the expression of *BrSAGs* and *BrORE1*. Our findings provide insight into the further understanding of mechanisms underlying senescence-induced SI breakdown and on the development of strategies to overcome SI for the propagation of parental inbred lines.

## Plant Materials and Methods

Heading Chinese cabbage double haploid “14 CR,” which is an *S*_46_ haplotype, and high-generation inbred line “6683” were from Henan Academy of Agricultural Sciences. Heading Chinese cabbage DHB848 is a commercial variety of Chinese cabbage. If not specified, 14 CR was used. Radish high-generation inbred line “Weixianqing 40” was from Shandong Academy of Agricultural Sciences. Flowering Chinese cabbage high-generation inbred line “Youqing 49 Caixin” was from Zhejiang University. All of these plants—Chinese cabbage, radish, and flowering Chinese cabbage—possess strong SI, with no more than five self-pollen tubes penetrating the stigma. Chinese cabbage seeds and radish seeds were germinated in pots of soil for 1 week, vernalized in ∼7°C for ∼1 month, and then planted in a greenhouse. No vernalization process is needed for flowering Chinese cabbage.

### Reproduction Phenotype Observation and Compatibility Analysis

For pollen tube observation in the senescent flowers of Chinese cabbage and radish, 0-d flowers from just-bolted plants (young) were emasculated, remained on the inflorescence for 0–6 days after emasculation (DAE), and pollinated with self- or cross-pollen. For pollen tube observation in flowers of young or old plants of Chinese cabbage and radish, 0-d flowers from just-bolted plants (young) or from plants passed their full-bloom stage (old) were emasculated and pollinated with self-pollen right away. Stigmas at 6 h after pollination (HAP) were fixed in Canoy’s fixative, softened in 10 M NaOH, and stained in 0.1% aniline blue. Pollen tubes were visualized by epifluorescence (Ex375-328/DM415/BA351p) on a Nikon Eclipse Ni and captured by a DS-Ri2 digital camera. Strong SI was defined as when stigmas were penetrated with 0–5 self-pollen tubes, and medium SI was defined as when stigmas were penetrated with 5–30 self-pollen tubes. Breakdown of SI or compatible response was defined as when stigmas were penetrated with more than 30 self- or cross-pollen tubes, respectively. The impact of senescence, chemicals, or related genes on compatibility was presented as number of pollen tubes that had penetrated the stigma.

Pistils at 12 days after pollination (DAP) were measured for pod length and counted for the number of seeds per pod. Strong SI was defined as when the average seed number was fewer than one per pod.

### Transient Gene Suppression Using Anti-sense Oligodeoxyribonucleotide (AS-ODN)

For transient gene suppressing using AS-ODN, stigmas mock treated or treated with S-ODN were used as control. Protocol followed that of Su et al. (2020). In brief, ∼1 mm of the unpollinated 0 DAE stigmas were cut and placed on the PGM medium (5 mM CaCl_2_, 5 mM KCl, 0.01% H_3_BO_3_, 1 mM MgSO_4_∙7H_2_O, 10% sucrose, pH 7.5, and 1% agarose) containing the corresponding S- or AS-ODN, or mock solution for 1 h, then pollinated with cross-pollen. The stigmas were maintained in a small well and kept in an incubator with 22.5°C/45% humidity. Treated stigmas were then fixed in Canoy’s fixative at 2 HAP, softened in 10 M NaOH, and stained in 0.1% aniline blue to check for pollen tube growth and PCD in the stigma.

S- or AS-ODN was designed based on the analysis by Sfold^1^) and RNAfold^2^, and BLAST program^3^ was used to assess potential off-target effect. To maintain ODN stability, 3 bases at both ends of S- or AS-ODN were phosphorothioate modified. ODNs were synthesized in Beijing Genomics Institution (BGI). At least three pairs of AS-ODN and S-OGN were tested, and sequences that are most effective are listed in Supplementary Table 2.

### Stigmatic Papilla Cell Death Detection

For the detection of PCD in the stigma, propidium iodide (PI) or trypan blue was used as cell death indicators. For PI staining, stigmas were stained in 50 μM PI (PBS, pH7.5) for 20 min, washed three times in PBS, and observed in the red channel (Ex 535 mm/BA 615 nm) on a Nikon Eclipse Ni, and images were captured by a DS-Ri2 digital camera. For the demonstration of nucleus change with the progression of senescence, PI-stained nuclei in 1–6 DAE stigmas were measured for area and also for the ratio of length/width. For trypan blue staining, 0 DAE stigmas and 4 DAE stigmas were stained in 0.01% trypan blue at 40°C for 30 min, washed in water three times, decolorized in Carnoy’s fixative for 3 days, and washed in water before observation under brightfield on a Nikon SMZ18, and images were captured by a DS-Ri2 digital camera. For the effect of self- or cross-pollination on PCD in the stigma, 0-d flowers were emasculated, remained unpollinated, or pollinated with self- or cross-pollen. For the effect of senescence on PCD, pistils at 0–4 DAE from young plants or 0 DAE pistils from young or old plants were kept unpollinated or pollinated with self-pollen. For the effect of AS-ODN on PCD, 0 DAE stigmas were mock treated or pretreated with S- or AS-ODN for 1 h and then pollinated with cross-pollen. Stigmas at 1 HAP were used for PI staining.

### Quantitative RT-PCR

Plant RNA Mini Kit (DP420, Tiangen) and the HiScript II Q RT SuperMix for qPCR (R222-01,Vazyme) were used for RNA extraction and reverse transcription. ChamQ SYBR qPCR Master Mix (Q711-03, Vazyme) and a qTOWER 3 qPCR machine (Analytikjena, Germany) were used for qRT-PCR, with BrACTIN 2 as the internal control. Primer sequences are listed in Supplementary Table 1.

Forty stigmas under different conditions were collected for the expression of related genes. For the effect of senescence, 0–3 DAE stigmas were collected. For the effect of self- or cross-pollination, 0 DAE stigmas were unpollinated or pollinated with self- or cross-pollen, and stigmas were collected at 1 h after pollination. For the effect of AS-ODN, 0 DAE stigmas were mock treated or pretreated with S- or AS-ODN for 1 h. For the effect of ethylene, unpollinated 0 DAE stigmas were cut at ∼3 mm from the top, placed on the PGM medium containing 10 mM ethephon, and treated in an incubator with 22.5°C/45% humidity for 5 h. Each set of qRT-PCR was repeated three times with comparable results.

### Ethephon Spray to Promote the Formation of Self-Fertilized Seeds

Inflorescences from young plants of flowering Chinese cabbage (*Brassica campestris* L. spp. *chinensis* var. *utilis Tsen et Lee*) and radish (*R. sativus*) were used for ethephon spray. Senescent flowers and the floral buds together with the shoot meristem were removed and only 0-d flowers remained on the inflorescences. Inflorescences were sprayed twice at a 30-min interval with different concentrations of ethephon supplemented with 0.0125% Tween-20 for better permeability. Inflorescences sprayed with water containing 0.0125% Tween-20 were used as control. When the stigmas were dried, which takes ∼1 h, pistils were pollinated with self-pollen grains and the inflorescences were put in mesh bags. Pod length and seed number per pod were recorded after 12 DAP.

### Data Analysis and Statistics

Each experiment was repeated at least three times with comparable results, shown as average ± S.D. in each histogram. For each histogram, *n* indicates the number of stigmas tested, the number of siliques tested, or the number of seeds tested. *P*-value was calculated using Student’s *t*-test, ^∗^ indicates *P* < 0.05 and ^∗∗^ indicates *P* < 0.01.

## Results

### Senescence Induces Breakdown of SI in Chinese Cabbage

**FIGURE 1:**
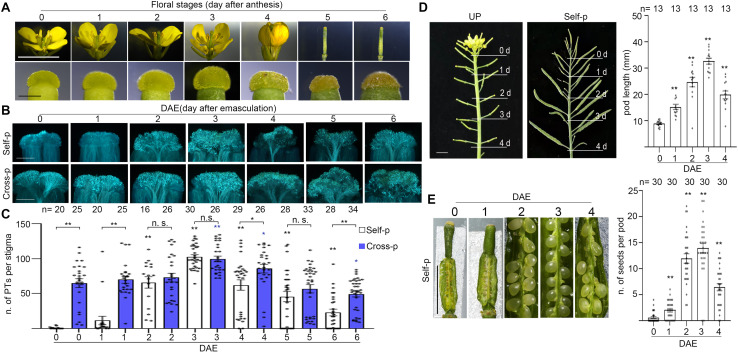
Flower senescence induces SI breakdown in Chinese cabbage. **(A)** Flowers at 0–6 days after anthesis (upper panel) and the corresponding stigmas (bottom panel). 0-d flower, just opened with indehiscent anthers; 1-d flower, 1 day after anthesis with dehiscent anthers; 2-d flower, 2 days after anthesis with wilted anthers; 3-d flower, 3 days after anthesis and flower starts to close; 4-d flower, 4 days after anthesis that are completely closed. 5-d and 6-d flowers only have a withered pistil and all other floral organs have fallen off, accompanied with collapsed papilla cells. **(B,C)**. Flowers at 0-d were emasculated and pollinated at 0–4 days after emasculation (DAE) with self- or cross-pollen. C shows the number of pollen tubes that penetrated each stigma. **(D)** An inflorescence with 0–4 DAE pistils (left image) were pollinated with self-pollen, and pod length was measured at 12 DAP (right image). **(E)** Pistils at 0–4 DAE were pollinated with self-pollen, and the number of seeds per pod was counted at 12 DAP. Scale bars = 1 cm (**A**, upper panel); 500 μm (**A**, lower panel); 500 μm **(B)**; 1 cm **(D,E)**. If not specified, * and **, significant (*P* < 0.05) and highly significant difference (*P* < 0.01) between self- or cross-pollinated 0 DAE stigmas and their counterparts of 1–6 DAE stigmas, respectively. n.s. indicates no significant difference. *, ** or n.s. above the bracket show comparisons of these samples. *n* indicates the number of stigmas or pods tested.

**FIGURE 2:**
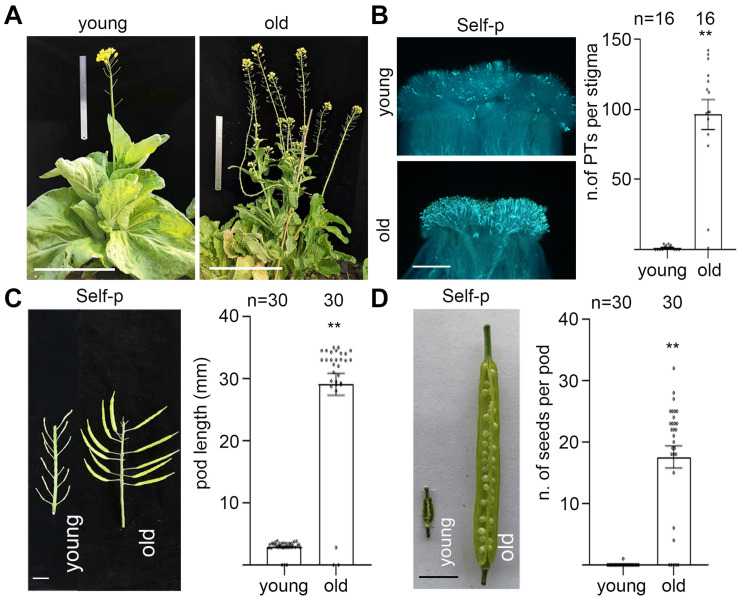
Plant senescence induces breakdown of SI in Chinese cabbage. **(A)** Young and old plants of Chinese cabbage. Supplementary Figure 1A shows all three varieties of Chinese cabbage. **(B)** Stigmas of 0-d flowers from young and old plants of Chinese cabbage were emasculated (0 DAE) and pollinated with self-pollen. The number of pollen tubes that penetrated each stigma is shown in the right panel. **(C,D)** Stigmas of 0-d flowers from young and old plants of Chinese cabbage were emasculated (0 DAE) and pollinated with self-pollen. Pod length **(C)** and seeds per pod **(D)** were observed at 12 DAP. Scale bars = 30 cm **(A)**; 500 μm **(B)**; 1 cm **(C,D)**. **, highly significant difference (*P* < 0.01). *n* indicates the number of stigmas tested, or the number of pods tested.

To examine the effect of senescence on SI in Chinese cabbage, we first characterized the stage of flowers and the stage of plants. In field and greenhouse experiments, flowers from Brassica family plants senesce within 1 day after cross-pollination, whereas flowers that have not been pollinated by compatible pollen last 2–5 days on the inflorescence even after they pass the prime pollination stage. Flowers that are just open and have indehiscent anthers are designated as 0-d flowers (Figure 1A). For experiments in this study, we emasculated 0-d flowers and allowed them to grow for another 0–6 days before pollination or analysis. For brevity, these pistils or stigmas were referred to as 0–6 DAE pistils or 0–6 DAE stigmas. To correlate with the naturally senescent flowers, we marked the 0-d flowers and observed their morphologies at 1–6 days after anthesis. As shown in Figure 1A, 1-d flowers have dehiscent anthers full of yellow pollen grains and anthers above the stigma; 2-d flowers have withering anthers and anthers as tall as the stigma; 3-d flowers have withered anthers and petals start to close; 4-d flowers are completely closed (Figure 1A); 5-d and 6-d flowers have a withered pistil and all other floral organs are abscised (Figure 1A). For the stage of plants, plants from early bolting stage to full-bloom stage were designated as young plants (Figure 2A and Supplementary Figures 1A,D) that have been growing for 35–65 days after vernalization under our growth conditions. Plants that have passed their full-bloom stage and have been growing for more than 65 days after vernalization but still bearing 0-d flowers were designated as old or senescent plants (Figure 2A and Supplementary Figure 1D). We also observed the morphology of flowers at 1–6 days after anthesis from old plants and found that they looked similar to that from young plants (Supplementary Figure 1A).

To examine whether flower senescence has any impact on the strength of SI, we pollinated the 0–6 DAE stigmas from young Chinese cabbage plants with self- or cross-pollen grains and counted the number of pollen tubes that had penetrated the stigma. We found that cross-pollinated 0–4 DAE stigmas were penetrated by comparable number of pollen tubes, suggesting that the stage difference in 0–4 DAE stigmas does not cause obvious difference in receiving compatible pollen tubes (Figures 1B,C). In the case of self-pollination, no self-pollen tubes grew or penetrated the 0 DAE stigmas, whereas significantly more self-pollen tube penetration of 1 DAE stigmas was already observed, and with the progress of senescence, the increase of self-pollen tubes became progressively more dramatic in the 2–4 DAE stigmas (Figures 1B,C). For example, ∼18, 60, and 100 self-pollen tubes penetrated the 1, 2, and 3 DAE stigmas (Figures 1B,C). These results suggest that SI was stringent in 0 DAE stigmas for self-pollen rejection, but was weakened to completely broken down in 1–4 DAE stigmas as self-pollen tubes were allowed to grow and penetrate the senescent stigmas. As senescence progresses further, the number of self-pollen tubes and cross-pollen tubes were both significantly reduced in 5 DAE stigmas (*P* < 0.0001) and 6 DAE stigmas (*P* < 0.0001) relative to their counterparts in 3 DAE stigmas (Figure 1B). This was accompanied by notably shrunk stigmas reflecting collapse of papilla cells in 5 and 6 DAE stigmas (Figure 1A), suggesting that the functional life span to receive pollen tubes has started to end in 5 and 6 DAE stigmas (Figure 1B). To test the effect of senescence on the formation of self-fertilized seeds in SI Chinese cabbage, we pollinated 0–4 DAE pistils from young plants with self-pollen grains and compared pod length and seed setting at 12 DAP. We found that 0 DAE pistils showed the shortest pods (Figure 1D) and almost no seeds in the pods (Figure 1E), an indication of robust SI in young pistils. In contrast, the pods became longer and had more seeds per pod with the progress of senescence in 1–3 DAE pistils (Figures 1D,E), but the pods became shorter and had fewer seeds in 4 DAE pistils than 2–3 DAE pistils (Figures 1D,E). Considering that there are only ∼30 ovules in each pistil, 2–4 DAE pistils have more than enough pollen tubes, whether self- or cross-pollen tubes, to fertilize each ovule in the pistil. However, some ovules in 4 DAE pistils might be too old to form seeds. We therefore did not record the self-fertilized seeds in the withered 5 and 6 DAE pistils, although a few self-pollen tubes managed to penetrate (Figures 1B,C).

To examine whether plant senescence has any impact on the strength of SI, we pollinated 0–5 DAE stigmas from old plants with self- or cross-pollen from young plants. Results showed that the number of self-pollen tubes and cross-pollen tubes in 0–4 DAE stigmas from old plants were all more than 60, except that neither self- nor cross-pollen tubes germinated on 5 DAE stigmas (Supplementary Figures 1B,C). In contrast to 0 self-pollen tubes in 0 DAE stigmas from young plants, the finding that there were more than 60 self-pollen tubes in 0 DAE stigmas from old plants prompted us to verify whether the breakdown of SI in 0 DAE stigmas from old plants is common in other varieties of Chinese cabbage. We emasculated 0-d flowers from three varieties of young and old Chinese cabbage plants (Figure 2A and Supplementary Figure 1D), pollinated them with self-pollen grains, and observed the number of self-pollen tubes that had penetrated each stigma. Results showed that while no self-pollen germinated or penetrated the 0 DAE stigmas from young plants, an average of 50–100 self-pollen tubes penetrated that from old plants in all three varieties of SI Chinese cabbage plants (Figure 2B and Supplementary Figure 1E). For the pod growth and seed formation, 0-d flowers from young and old plants of three varieties of Chinese cabbage were emasculated and pollinated with self-pollen, and the pod length and seed setting were compared at 12 DAP. Similar to our findings in senescent flowers, self-pollinated pistils from old plants showed significantly longer pods and many more seeds per pod than those from young plants in all three varieties of Chinese cabbage (Figures 2C,D and Supplementary Figures 1F,G). Taken together, our results suggest that flower senescence and plant senescence behave similarly in breaking down SI in Chinese cabbage.

### Senescence Leads to PCD in Stigmatic Papilla Cells in Chinese Cabbage

**FIGURE 3:**
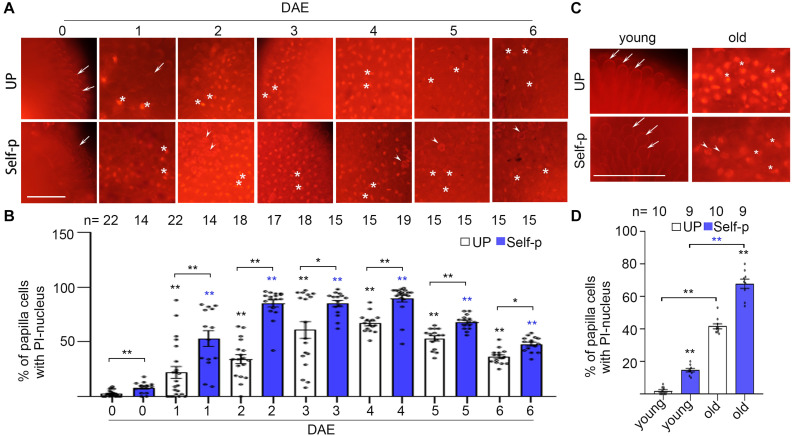
Senescence causes PCD in stigmatic papilla cells of Chinese cabbage. **(A,B)** Papilla cell death of 0–6 DAE stigmas. Stigmas were unpollinated or pollinated with self-pollen and stained in PI at 60 min after pollination. The whole stigma is shown in Supplementary Figure 2B. **(C,D)** Papilla cell death in 0 DAE stigmas from young and old plants. Stigmas were unpollinated or pollinated with self-pollen and stained in PI at 60 min after pollination. All three varieties of Chinese cabbage are shown in Supplementary Figures 2C–E. Scale bars = 100 μm. In **(A)** and **(C)**, arrows, PI-stained cell wall of papilla cells; white stars, PI-stained nuclei of papilla cells; arrowheads, pollen grains. If not specified, * and **, significant (*P* < 0.05) and highly significant difference (*P* < 0.01) between unpollinated or self-pollinated 0 DAE stigmas and their counterparts of 1–6 DAE stigmas, respectively. * or ** above the bracket show comparisons of these samples. *n* indicates the number of stigmas tested.

We previously found in Chinese cabbage that PCD in stigmatic papilla cells is induced by and required for cross-pollination but is abrogated in self-pollinated stigmas (Su et al., 2020; Supplementary Figure 2A). We therefore tested whether PCD is involved in senescence-associated breakdown of SI in Chinese cabbage. We first tested the compromise of plasma membrane integrity, which is a morphological feature of PCD (Kabbage et al., 2017). PI was used as a cell death indicator, with PI-stained nuclei reflecting plasma membrane permeability in dead papilla cells (Su et al., 2020). We found that the percentage of PI-stained nuclei was lower than 5% in unpollinated 0 DAE stigmas, whereas it increased to 20% and 60% in 1 and 3 DAE stigmas, respectively (Figures 3A,B and Supplementary Figure 2B). Another cell death indicator, trypan blue, showed that ∼45% papilla cells in 4 DAE stigmas were stained blue whereas no blue papilla cells were found in 0 DAE stigmas (Supplementary Figure 3A). Altogether, our finding that the increasing number of dead papilla cells in 0–3 DAE stigmas was in line with the increasing number of self-pollen tubes in 0–3 DAE stigmas suggests that water and other factors might leak out of the porous papilla cells to support the germination and growth of self-pollen. Meanwhile, the comparable number of cross-pollen tubes in 0–3 DAE stigmas (Figure 1B) suggests that PCD in 0–3 DAE stigmas does not significantly compromise the receptivity of the stigma.

The change of nucleus is another hallmark of PCD that reflects the progress of PCD. We therefore observed the shape change of nucleus among the PI-stained nuclei in 1–6 DAE stigmas, to prove that PCD in the papilla cells of senescent stigmas is an irreversible process. In 1 DAE stigmas, which is at the early stage of PCD, the PI-stained nuclei were usually round (Figure 3A). With the progress of senescence, the nuclei became irregular, presented a bar-like shape in 2–4 DAE stigmas, and became crescent-shaped and distorted in 5–6 DAE stigmas (Figure 3A). We then quantified the change of nucleus by measuring the area, or calculating the ratio of length/width of each PI-stained nucleus in 1–6 DAE stigmas. Supplementary Figures 3D–F showed that from 1 DAE to 6 DAE stigmas, the nucleus became smaller and slenderer, especially in 5–6 DAE stigmas, suggesting that the nucleus damage become more serious with the progress of senescence. In addition, the number of PI-stained nucleus was reduced in 5 DAE (*P* < 0.05) and 6 DAE (*P* < 0.0001) stigmas compared to that in 4 DAE stigmas (Figure 3B), suggesting that the degradation of nucleus occurred in 5 DAE and 6 DAE stigmas. Altogether, our results demonstrated that PCD in the papilla cells of 0–6 DAE stigmas was an irreversible process.

The finding that papilla cells with PI-stained nucleus were still capable to support pollen tube growth led us to use the viability probe, fluorescein diacetate (FDA), to test the viability of the cells in 0–6 DAE stigmas. FDA is a cell-permeant esterase substrate that requires intracellular esterases to activate its fluorescence and also requires cell-membrane integrity for cellular retention. We found that the number of FDA-negative papilla cells was progressively increasing in 0–6 DAE stigmas (Supplementary Figures 3B,C), which is not exactly the same compared to the change of cells with PI-stained nucleus (Figures 3A,B). For example, the number of FDA-negative cells increased from 0% in 0–1 DAE stigmas to < 10% in 2–3 DAE stigmas and further increased to 20–55% in 4–6 DAE stigmas (Supplementary Figures 3B,C), whereas the number of cells with PI-stained nucleus increased from 0% in 0 DAE stigmas to 40–60% in 2–4 DAE stigmas but decreased to 60–40% in 5–6 DAE stigmas (Figure 3B). Although the stigmas were not dual-stained with PI and FDA and individual cells were not observed by time-course confocal microscopy, the PI pattern and FDA pattern in 0–6 DAE stigmas suggest that many of the papilla cells in 1–3 DAE stigmas showed both cytoplasmic fluorescein and nuclear PI. This result is similar to rice blast invasion-caused damage to the host cells, in which both FDA and PI are positive in newly invaded cells but fully invaded cells only have PI-positive nucleus (Jones et al., 2016). We speculate at this stage of senescence, such as 0–3 DAE stigmas, that some papilla cells were in the process of cellular dismantling with the loss of membrane integrity, gradually allowing both PI to enter and fluorescein to diffuse out. This also explains our result that 1–3 DAE stigmas with PI-stained nucleus still support pollen tube growth. However, with the progress of senescence such as in 4–6 DAE stigmas, the papilla cells may become more porous and lost cellular retention of FDA as well as the receptivity to support pollen germination and growth. These results provided rational explanations for the reduced number of self- or cross-pollen tubes in 4–6 DAE stigmas than that in 3 DAE stigmas (Figures 1B,C) and for the reduced number of self-fertilized seeds in 4 DAE pistils than that in 2–3 DAE pistils (Figure 1E). Altogether, our results suggest that the progress of PCD in senescent stigmas is an irreversible process, from the compromise of plasma membrane integrity in 1–3 DAE stigmas to deformed nucleus in 4–6 DAE stigmas.

Furthermore, we found that self-pollination triggered more papilla cell death in senescent stigmas (Figures 3A,B and Supplementary Figure 2B). For example, the percentage of dead papilla cells in unpollinated 1 DAE and 2 DAE stigmas was ∼25% and ∼40%, respectively, but the percentage increased to ∼52% and ∼90% after self-pollination (Figure 3B). It was likely that some of the papilla cells of the unpollinated senescent stigmas did not show PI-stained nuclei, but the strength of SI was already compromised; therefore, self-pollination triggered more PCD in these senescent stigmas (Figure 3B), consistent with compatible pollination-triggered PCD in papilla cells (Su et al., 2020, also shown in Supplementary Figure 2A).

Similarly, we compared the percentage of papilla cell death in 0 DAE stigmas from young and old plants in three varieties of SI Chinese cabbage. The percentage of dead papilla cells, indicated by PI-stained nuclei, was significantly higher in unpollinated 0 DAE stigmas from old plants than that from young plants in all three varieties of Chinese cabbage plants, and self-pollination further promoted cell death (Figures 3C,D and Supplementary Figures 2C–E). Together, these results suggest that senescence mimics the effect of cross-pollen (Su et al., 2020) in causing PCD in stigmatic papilla cells and therefore breaks down SI in senescent flowers and young flowers from senescent plants before pollination.

### Senescence-Associated Genes Are Required for Papilla Cell PCD

To investigate whether the breakdown of SI in senescent stigmas is simply due to age-dependent reduction of SRK expression or senescence-associated PCD in the papilla cells, we first examined the expression level of SRK in 0–4 DAE stigmas. As shown in Supplementary Figure 4, the level of SRK transcripts in 1 DAE stigmas was not different from that in 0 DAE stigmas, but it declined to ∼60% in 2 DAE stigmas and only ∼10% in 3 DAE stigmas and 4 DAE stigmas. However, the breakdown of SI started to occur in 1 DAE stigmas (Figures 1B,C), which is before the reduction of SRK expression. Our result is consistent with the report that in older *Brassica* flowers, SRK transcript and protein level decline, but they usually remain above the threshold required for maintenance of the SI response (Nasrallah et al., 2002). However, 1 DAE stigmas had ∼20% PI-positive papilla cells, and allowed the growth of ∼10 self-pollen tubes. Altogether, the results that the breakdown of SI and PCD both occurred in 1 DAE stigmas suggest that papilla cell PCD is closely correlated to the breakdown of SI in senescent stigmas.

**FIGURE 4:**
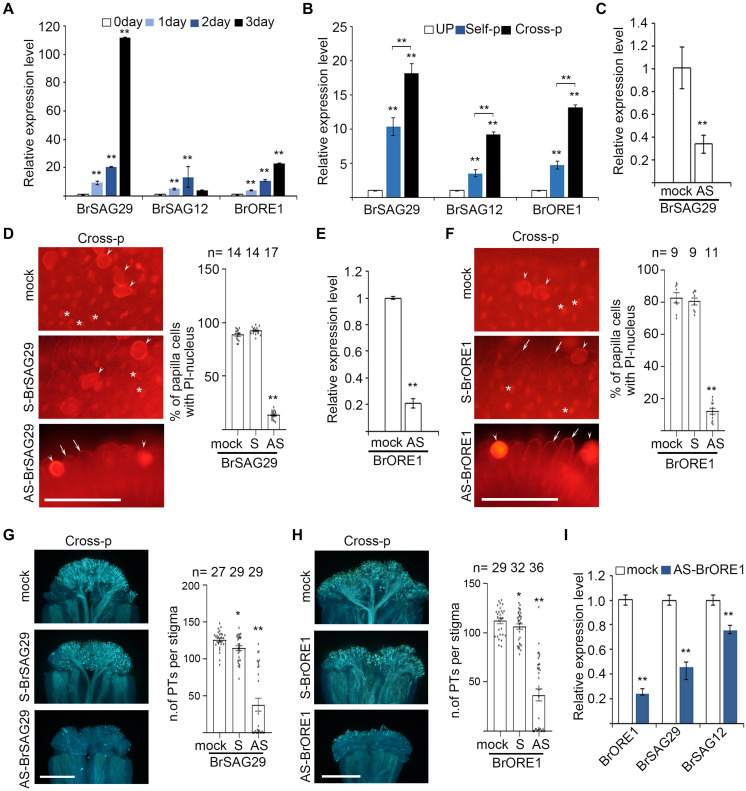
Senescence-associated genes are upregulated in senescent flowers and are essential for papilla cell PCD. **(A,B)** Quantitative RT-PCR analysis of *Brassica rapa* senescence-associated genes in 0- to 3-day unpollinated stigmas **(A)** and in unpollinated, self-pollinated, or cross-pollinated stigmas **(B)**. Nomenclature of *BrSAG29*, *BrSAG12*, and *BrORE1* are based on their *Arabidopsis thaliana* homologs. Primers used for qRT-PCR are listed in Supplementary Table 1. **(C,E)** AS-ODN targeting *BrSAG29* **(C)** and *BrORE1* **(E)** suppress the expression of corresponding genes in young unpollinated stigmas. **(D,F)** PI staining of cross-pollinated stigmas that were pretreated with mock, S- or AS-BrSAG29, and BrORE1, respectively. Full image is shown in Supplementary Figures 5C,D. **(G,H)** Pollen tube growth and penetration in cross-pollinated stigmas that were pretreated with mock, S-, or AS-BrSAG29 and BrORE1, respectively. **(I)** The expression of *BrSAG12* and *BrSAG29* in stigmas pretreated with AS-BrORE1. Scale bars = 100 μm **(D,F)**; 500 μm **(G,H)**. In **(D,F)**, arrows, PI-stained cell wall of papilla cells; white stars, PI-stained nucleus of papilla cells; arrowheads, pollen grains. * and **, significant difference (*P* < 0.05), and highly significant difference (*P* < 0.01), respectively. *n* indicates the number of stigmas tested.

We therefore studied how PCD is regulated in senescent stigmas and whether disturbing these regulators affect pollen–stigma interactions. Among the senescence-related genes we analyzed (Supplementary Figures 5A,B), *BrSAG29*, *BrSAG12*, and *BrORE1* were upregulated in unpollinated senescent stigmas (Figure 4A). Interestingly, the expression of these genes was also significantly (*P* < 0.01) higher in cross-pollinated stigmas than that in self-pollinated stigmas (Figure 4B), suggesting that senescence-associated PCD and cross-pollination-triggered PCD are likely to be regulated similarly by these genes.

We aimed to study the function of *BrSAG29*, *BrSAG12*, and *BrORE1* in papilla cell death, but an efficient transformation protocol for heading Chinese cabbage is not available. Antisense oligodeoxyribonucleotide (AS-ODN) has been shown to function as a powerful tool to suppress genes in *in vitro* grown pollen tubes (Liao et al., 2013; Mizuta and Higashiyama, 2014; Chai et al., 2017; Chen et al., 2018) and in the stigma feeding assay that we developed to manipulate the stigma environment and examine the effect on pollen germination and penetration (Duan et al., 2014; Su et al., 2020). We therefore used AS-ODN to examine if suppressing the expression of *BrSAG29* or *BrORE1* would affect the growth and penetration of compatible pollen tubes in 0 DAE stigmas. As shown in Figures 4C,E, the abundance of *BrSAG29* transcript and *BrORE1* transcript was significantly reduced in stigmas treated with the corresponding AS-ODN than that in mock-treated stigmas. Cross-pollination triggered 80–90% of papilla cell death in stigmas pretreated with S-BrSAG29 or S-BrORE1 and in mock-treated stigmas (Figures 4D,F). However cross-pollination-triggered papilla cell death occurred only ∼10% in stigmas pretreated with AS-BrSAG29 or AS-BrORE1 (Figures 4D,F). Meanwhile, the growth and penetration of cross-pollen tubes were significantly inhibited in stigmas pretreated with AS-BrSAG29 or AS-BrORE1, which were both only ∼1/4 of that in mock-treated stigmas or stigmas pretreated with S-BrSAG29 or S-BrORE1 (Figures 4G,H). These results suggest that *BrSAG29* and *BrORE1* are essential for papilla cell PCD and compatibility responses.

To explore whether *BrSAGs* are regulated by *BrORE1* in papilla cell death, similar to that in the senescence of Arabidopsis leaf or flower (Wagstaff et al., 2009), we treated 0 DAE stigmas with AS-BrORE1 and detected the expression of *BrSAGs*. Results showed that not only *BrORE1* but also *BrSAG29* and *BrSAG12* were significantly suppressed (Figure 4I), suggesting that *BrORE1* functions upstream of *BrSAG29* and *BrSAG12* in regulating the senescence of stigma. Altogether, these results suggest that senescence upregulates the *BrORE1* transcription factor, promotes the expression of *BrSAGs* such as *BrSAG29* and *BrSAG12*, and triggers PCD in papilla cells of senescent stigmas or stigmas of young flowers from senescent plants.

### Ethylene Is Involved in the Senescence of Chinese Cabbage Stigmas

We previously reported that ethylene response is upregulated in cross-pollinated stigmas and ethylene promotes PCD in papilla cells and breaks down SI in Chinese cabbage (Su et al., 2020). We also found in this study that stigma senescence was regulated by *BrORE1* (Figure 4I), which is a known transcription factor downstream of ethylene signaling in leaf senescence (Kim et al., 2009, 2014). These results prompted us to test whether senescence-associated PCD in the papilla cells is regulated by ethylene.

**FIGURE 5:**
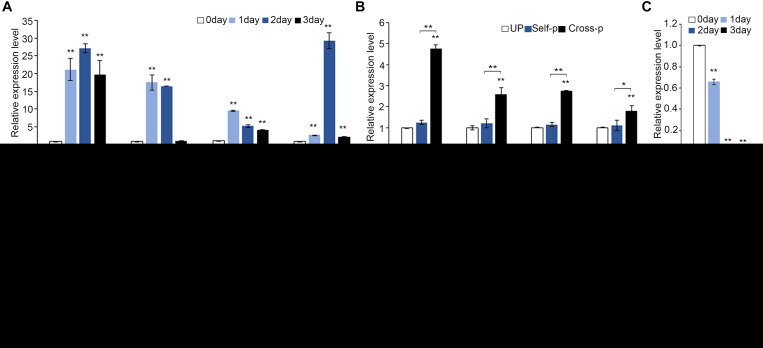
Ethylene is involved in the senescence of Chinese cabbage stigmas. **(A)** The expression of *Brassica rapa* ACC synthase genes (*BrACSs*) in senescent stigmas. **(B)** The expression of *Brassica rapa* ACC synthase genes (*BrACSs*) in stigmas unpollinated or pollinated with self- or cross-pollen. **(C)** The expression of *BrCTR1* in senescent stigmas. **(D)** The expression of *BrERFs* in senescent stigmas. **(E)** The expression of ethylene response genes and senescence-associated genes in stigmas treated with ethephon. **(F)** The expression of senescence-associated genes in stigmas treated with AS-BrERF012.1. **, indicate highly significant difference compared with the corresponding white column. * or ** above the bracket show comparisons of these samples.

We first analyzed the expression pattern of genes involved in ethylene biosynthesis in 0–4 DAE stigmas of Chinese cabbage. The rate-limiting factor in ethylene production is considered to be the synthesis of 1-aminocyclopropane-1-carboxylic acid (ACC), the immediate precursor of ethylene (Yamagami et al., 2003; Lee and Yoon, 2018; Marczak et al., 2020). Genes in *B. rapa* were named based on their homologs in *A. thaliana*. Among the ACC synthase genes in *B. rapa* (*BrACSs*) that we analyzed (Supplementary Figures 6A,B), *BrACS6*, *BrACS9.2*, *BrACS10*, and *BrACS12* were greatly upregulated in senescent stigmas (Figure 5A). These four *BrACS* genes were also upregulated in cross-pollinated stigmas, but their expression remained low in unpollinated stigmas and in self-pollinated stigmas (Figure 5B).

We next analyzed the expression pattern of genes involved in ethylene response in 0–4 DAE stigmas of Chinese cabbage. Constitutive triple response 1 (*CTR1*) and ethylene response factors (*ERFs*) in *A. thaliana* play a negative and a positive regulatory role in ethylene signaling pathway, respectively (Dubois et al., 2018; Binder, 2020; Su et al., 2020). Among the *BrCTR1* and *BrERFs* that we analyzed (Supplementary Figure 5C), the expression of *BrCTR1* was suppressed or totally abrogated in senescent stigmas (Figure 5C), and the expression of *BrERFs*, such as *BrERF012.1*, *BrERF109*, *BrERF017*, and *BrERF012.2*, was upregulated (Figure 5D). These results are in line with our previous findings that AS-BrCTR1 treatment induces papilla cell PCD and the breakdown of SI and that AS-ERF012.1 treatment suppresses papilla cell PCD and the growth of compatible pollen (Su et al., 2020).

To further investigate the involvement of ethylene in flower senescence in Chinese cabbage, we tested whether increasing ethylene in 0 DAE stigmas affects the expression of *BrORE1* and *BrSAG*s. As shown in Figure 5E, the expression of *BrCTR1* was significantly suppressed and *BrERF012.1* was significantly induced in ethephon-treated stigmas. More importantly, the expression of *BrORE1*, *BrSAG12*, and *BrSAG29* was drastically upregulated in ethephon-treated stigmas (Figure 5E). We also examined whether blocking ethylene response by suppressing *BrERF012.1* affects the expression of these genes. Results showed that the transcripts of *BrORE1*, *BrSAG29*, and *BrSAG12* were greatly reduced in unpollinated 0 DAE stigmas treated with AS-BrERF012.1 than that in mock-treated stigmas (Figure 5F). Together, these results are consistent with ethylene being an important component in cross-pollination-triggered PCD and senescence-associated PCD in papilla cells.

### Papilla Cell PCD Is Associated With Senescence-Induced SI Breakdown in Radish

Radish belongs to the genus *Raphanus*, but its SI is similarly controlled as in the Brassica plant Chinese cabbage and also uses SI for hybrid seed production. We therefore examined if the strength of SI in radish is also affected by senescence and whether PCD is involved.

**FIGURE 6:**
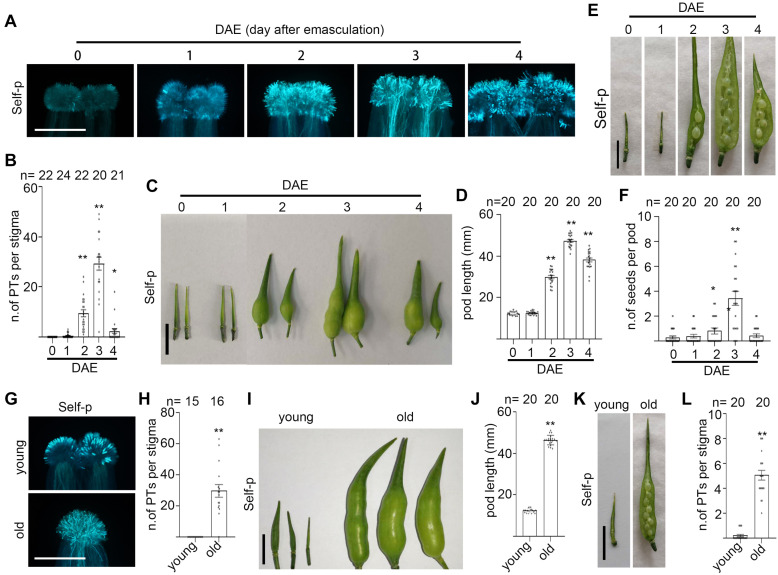
Senescence leads to the breakdown of SI in radish plants. **(A,B)** The growth and penetration of self-pollen in 0–4 DAE radish stigmas. **(C,D)** Pod length of radish 0–4 DAE pistils at 12 DAP. **(E,F)** Seed set of radish 0–4 DAE pistils at 12 DAP. **(G,H)** The growth and penetration of self-pollen in 0 DAE pistils from young or old radish plants. **(I,J)** Pod length of 0 DAE pistils from young or old radish plants at 12 DAP. **(K,L)** Seed set of 0 DAE pistils from young or old radish plants at 12 DAP. Scale bars = 500 μm **(A,G)**; 1 cm **(C,E,I,K)**. * and **, significant difference (*P* < 0.05), and highly significant difference (*P* < 0.01), respectively. *n* indicates the number of stigmas tested or the number of pods tested.

To test the effect of senescence on the strength of SI in radish, we staged radish flowers based on the days after anthesis, similar to Chinese cabbage flowers. Control radish flowers were marked at 0 d, remained not emasculated, and grew for another 0–4 days to correlate with the natural senescent stages (Supplementary Figure 7A). For experiments, 0-d flowers were emasculated, waited for 0–4 days (0–4 DAE), and then pollinated with self-pollen grains. As shown in Figures 6A,B, 0 DAE stigmas had no self-pollen tubes penetrated, whereas increasingly more self-pollen tubes penetrated 2–3 DAE stigmas. Consequently, 2–3 DAE pistils showed longer pod and more seeds per pod than 0 DAE pistils at 12 DAP (Figures 6C–F). We also found that 4 DAE stigmas have fewer self-pollen tubes and the pods were shorter and produced fewer self-fertilized seeds (Figures 6A–F), probably due to senescence-caused decrease in stigma receptivity. Similarly, 0 DAE stigmas from old plants allowed the penetration of more than 30 self-pollen tubes (Figures 6G,H), the pods were longer, and seeds per pod were more than that from young plants (Figures 6I–L). These results together suggest that senescence compromised the strength of SI in radish.

**FIGURE 7:**
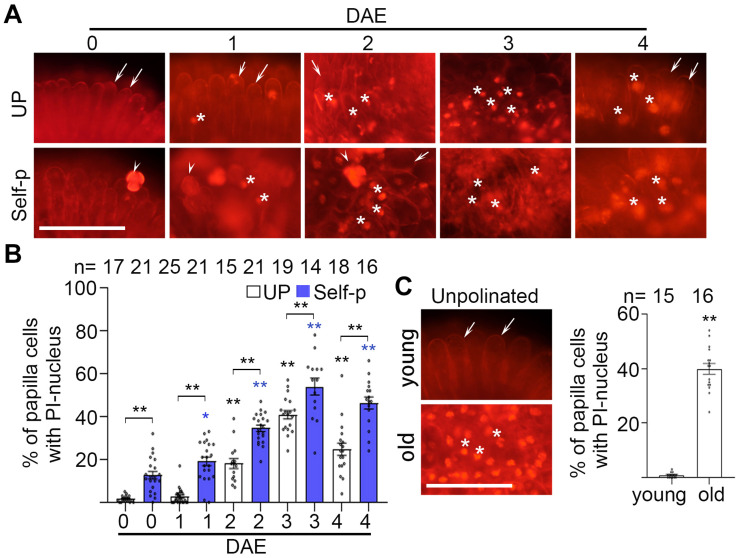
Senescence causes PCD in radish plants. **(A,B)** Papilla cell death in 0–4 DAE radish stigmas. Stigmas at 0–4 DAE were unpollinated or pollinated with self-pollen and stained in PI at 60 min after pollination. The image of each stigma was magnified from the rectangular outlined area shown in Supplementary Figure 8A. **(C)** Papilla cell death in 0 DAE stigmas from young and old radish plants, indicated by PI staining. The image of each stigma was magnified from the rectangular outlined area shown in Supplementary Figure 8B. Scale bars = 100 μm **(A,C)**. In **(A,C)**, arrows, PI-stained cell wall of papilla cells; white star, PI-stained nucleus of papilla cells; arrowhead, pollen grains. * and **, significant difference (*P* < 0.05), and highly significant difference (*P* < 0.01), respectively. ** above the bracket shows comparisons of these samples. *n* indicates the number of stigmas tested.

We next investigated if PCD is also involved in senescence-associated breakdown of SI in radish. Consistent with our findings in senescent Chinese cabbage flowers (Figures 3, 4), senescent radish flowers showed increasingly higher percentage of dead papilla cells with the progress of senescence, indicated by PI-stained nuclei (Figures 7A,B and Supplementary Figure 8A). For example, ∼40% dead papilla cells were found in 3 DAE stigmas whereas ∼0% was found in 0 DAE stigmas (Figures 7A,B). Furthermore, self-pollination aggravated the severity of PCD; for example, the percentage of dead papilla cells were increased from 40% to 55% after self-pollination (Figures 7A,B). Similarly, 0 DAE stigmas from old plants showed ∼40% dead papilla cells whereas that from young plants had very low level of papilla cell death (Figure 7C and Supplementary Figure 8B). Similarly, dead papilla cells indicated as FDA-negative were progressively increasing in 0–6 DAE stigmas of radish plants (Supplementary Figures 8C,D). Taken together, these findings show that senescence-induced PCD may underlie the SI breakdown in Chinese cabbage and in radish.

### Spraying Ethephon Promotes the Formation of Self-Fertilized Seeds in Flowering Chinese Cabbage and Radish

**FIGURE 8:**
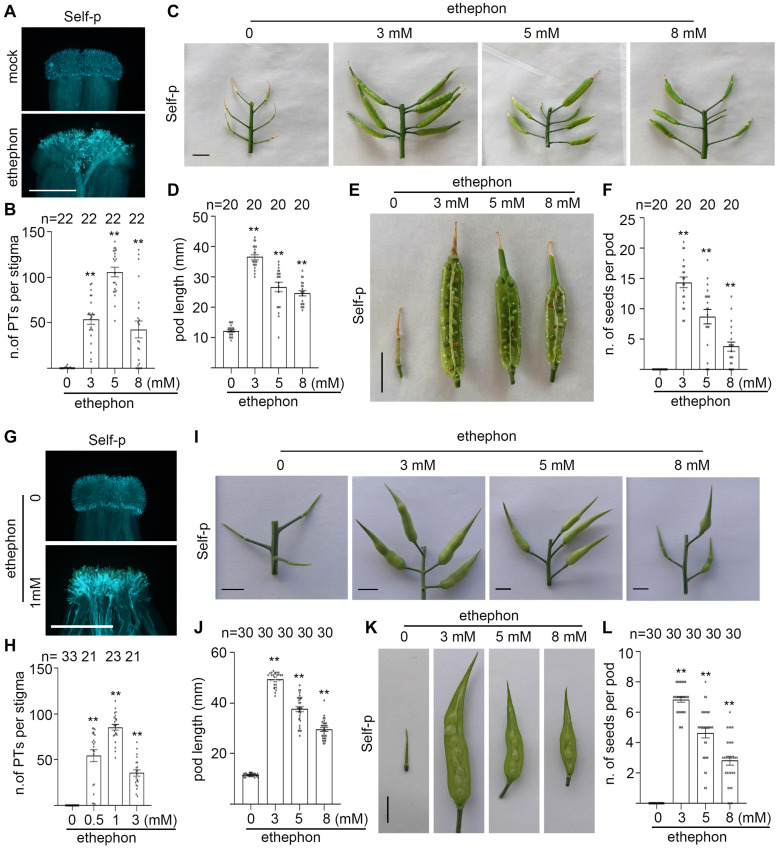
Spraying ethephon promotes the formation of self-fertilized seeds in flowering Chinese cabbage and in radish. **(A,B)** The growth and penetration of self-pollen tubes in flowering Chinese cabbage stigmas with or without ethephon spray, followed by self-pollination. **(C,D)** Pod length of flowering Chinese cabbage inflorescences with or without ethephon spray, followed by self-pollination. Pods were measured at 20 DAP. **(E,F)** Seed setting of flowering Chinese cabbage inflorescences with or without ethephon spray, followed by self-pollination. Seeds per pod were counted at 20 DAP. **(G,H)** The growth and penetration of self-pollen tubes in radish stigmas with or without ethephon spray, followed by self-pollination. **(I,J)** Pod length of radish inflorescences with or without ethephon spray, followed by self-pollination. Pods were measured at 15 DAP. **(K,L)** Seed setting of radish inflorescences with or without ethephon spray, followed by self-pollination. Seeds per pod were counted at 15 DAP. Scale bar = 500 μm **(A,G)**; 1 cm **(C,E,I,K)**. Arrows, PI-stained cell wall of papilla cells; star, PI-stained nucleus of papilla cells; arrowhead, pollen grains. * and **, significant difference (*P* < 0.05), and highly significant difference (*P* < 0.01), respectively. *n* indicates the number of stigmas tested or the number of pods tested.

The ethylene-releasing reagent, ethephon, is widely used to promote ripening of many fruits (Ampa et al., 2017) or to increase the percentage of female flowers in Cucumis family vegetables (Hao et al., 2003; Duan et al., 2008; Wang et al., 2010). We showed previously that spraying ethephon overcomes SI in heading Chinese cabbage (Su et al., 2020). Considering the similarity between radish and Chinese cabbage in PCD and the breakdown of SI in senescent flowers and senescent plants, we hypothesize that spraying ethephon overcomes SI and promotes the formation of self-fertilized seeds in flowering Chinese cabbage and SI radish. To test our hypothesis, we sprayed the inflorescences of flowering Chinese cabbage plants and inflorescences of radish plants with different concentrations of ethephon, pollinated with self-pollen, and observed the number of self-pollen tubes that penetrated the stigma at 6 HAP. Results showed that flowering Chinese cabbage stigmas and radish stigmas from the ethephon-sprayed inflorescences allowed the penetration of bundles of self-pollen tubes (Figures 8A,B,G,H), whereas no self-pollen tubes penetrated stigmas from mock-sprayed inflorescences (Figures 8A,B,G,H). To observe the effect of spraying ethephon on the formation of self-fertilized seeds in flowering Chinese cabbage and in radish plants, the ethephon-sprayed inflorescences were pollinated with self-pollen and the pods were observed at 15 DAP for radish and at 20 DAP for flowering Chinese cabbage. As shown in Figure 8, the pods from ethephon-sprayed inflorescences of flowering Chinese cabbage plants and of radish plants both were significantly longer (Figures 8C,D,I,J) and had many more seeds per pod (Figures 8E,F,K,L) than those from mock-treated inflorescences. For example, inflorescences sprayed with 3 mM ethephon produced ∼15 seeds per pod in flowering Chinese cabbage and ∼7 seeds per pod in radish, whereas mock-treated inflorescences produced no seeds in these two strong SI plants (Figures 8E,F,K,L). Furthermore, we examined if spraying ethephon affects the viability of seeds. Seeds from ethephon-sprayed radish plants were germinated and the germination rate was compared with seeds from bud pollination, i.e., pollination carried out on the stigmas at their bud stages when SI system is not active. Results showed that the germination rate of seeds from ethephon-sprayed plants was close to 100%, as good as that of seeds from bud pollination (Supplementary Figure 9). Together, our results suggest that spraying ethephon overcomes SI and promotes the formation of self-fertilized seeds in flowering Chinese cabbage and radish.

## Discussion

**FIGURE 9:**
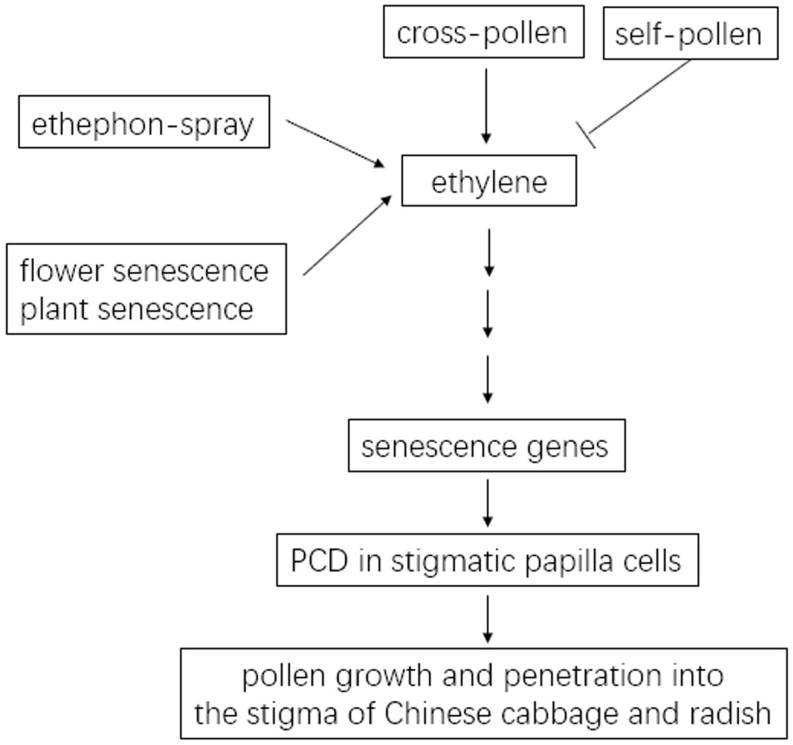
Model for the association of papilla cell PCD with senescence-induced SI breakdown in the Chinese cabbage and radish. In Chinese cabbage, cross-pollen upregulates ethylene signaling, causes PCD in stigmatic papilla cells, and results in the growth and penetration of cross-pollen into the stigma. These processes are abrogated in self-pollinated stigmas. In Chinese cabbage and in radish, senescence mimics the effect of cross-pollen in upregulating ethylene signaling and causing PCD in stigmatic papilla cells, which is associated with the breakdown of SI in senescent flowers and in senescent plants. Spraying the ethylene-releasing reagent, ethephon, on inflorescences breaks down SI of Chinese cabbage and radish.

Pollen–stigma interaction is the first checkpoint in fertilization to facilitate the germination and penetration of compatible pollen and the rejection of SI pollen or unrelated foreign pollen (Jany et al., 2019). For many SI plants, outcrossing introduces genetic diversity but self-fertilization ensures the production of progeny generations. The regulation of these two seemingly antagonistic but important processes is crucial for plant survival and for the breeding industry. It has been suggested that senescent stigmas have much weakened SI in transformed SI Arabidopsis Col-0 plants (Nasrallah et al., 2002, 2004; Tantikanjana and Nasrallah, 2012). However, how senescence affects the hybrid seed production and the propagation of SI parental lines had remained poorly understood. Therefore, it is important to uncover the molecular mechanism on how senescence affects SI at the level of the stigma as the earliest interface for pollination. Here, as summarized in Figure 9, PCD in the papilla cells of senescent stigmas in Chinese cabbage and in radish, is associated with the breakdown of SI and the formation of self-fertilized seeds.

Our findings that senescence-associated PCD demonstrated here and compatible pollination-induced PCD (Sankaranarayanan et al., 2013; Su et al., 2020) share similar functions and a common regulatory mechanism represent an important step forward for our understanding of stigmatic control of pollen germination, tube growth, and penetration. We establish that PCD in stigmatic papilla cells is essential and sufficient for pollen tube growth and penetration in senescent stigmas and in compatible pollination (Figures 2–4; Su et al., 2020). For example, the percentage of PI-stained nuclei increased from 5% in 0 DAE stigmas to 60% in 3 DAE stigmas (Figures 3A,B and Supplementary Figure 2B), similar to the level of cell death in stigmas at 30 min after cross-pollination when compatible pollen starts to germinate (Su et al., 2020; Supplementary Figure 2A). Since PI is not permeant to live cells, PI-stained nuclei reflect the permeability of plasma membrane and indicate the leak of water and other factors out of the papilla cells. Our finding that stigmas of senescent flowers or senescent plants have PI-stained nuclei in the papilla cells before pollination, mimicking the effect of cross-pollination, suggests that papilla cells with porous plasma membrane cannot seal water and other factors inside; therefore, they are capable of supporting the germination and tube growth of either self-pollen or cross-pollen. PCD in compatible pollination and in senescent stigmas also share regulatory components. We demonstrate that they are both regulated by *BrSAG12*, *BrSAG29*, and *BrORE1* (Figure 4) and that stigma senescence is dependent on ethylene signaling (Figures 5C–F). For example, ethephon-released ethylene induces the expression of *BrSAGs* and *BrORE1* while suppressing ethylene response by AS-BrERF012.1 downregulates the expression of *BrSAGs* and *BrORE1* (Figure 5F). In Su et al. (2020), we demonstrated that suppressing PCD by the caspase-inhibitor, Z-VAD-FMK, or suppressing the expression of regulatory genes of papilla cell PCD effectively inhibits compatible pollination, whereas inducing PCD by treating stigmas with ethephon effectively breaks down SI and supports the germination and growth of self-pollen. Taken together, our findings reported here and in Su et al. (2020) suggest that stigmatic papilla cell PCD, triggered by cross-pollination or by senescence, probably facilitate pollen hydration and germination by secreting water and other factors out of the damaged papilla cells. The lack of papilla cell PCD after self-pollination indicates that papilla cells reject self-pollen by preventing an ethylene-mediated PCD process (Su et al., 2020). It will be interesting to investigate in the future how self-pollen and cross-pollen selectively modify ethylene-mediated PCD to achieve the SI and SC response.

In a broader context, ethylene and PCD are often found involved in biotic or abiotic stresses (Hinz et al., 2010; Huysmans et al., 2017; Nascimento et al., 2018; Xiao et al., 2020; Noman et al., 2020). These environmental stresses such as high salinity, low light intensity, drought, pathogen attack, and nutrient deficiency promote premature senescence (Balazadeh et al., 2010; Zhao et al., 2016; Gao et al., 2018; Suzuki and Katano, 2018; Aacute et al., 2020; Hu et al., 2020; Tian et al., 2020). Therefore, these environmental stresses may cause the upregulation of ethylene signaling and the induction of PCD in papilla cells and are likely to reduce or completely break down SI in many Brassica family plants.

The ability to compromise or fully break down SI under unfavorable conditions such as senescence is vital from the perspective of the plants. For many Brassica family plants, outcrossing is preferred for the fitness of the progenies and selfing can result in inbreeding depression. Over a season, plant condition, pollinator availability, and environmental factors vary. When flowers have wasted more than one day waiting for outcrossing or plants have passed their full-bloom stage, it would seem strategically sensible for senescent flowers or senescent plants to opt for releasing the inhibition on self-pollen and produce some self-fertilized seeds rather than keeping stringent SI and producing no seed at all. Furthermore, since plants are sessile and have to survive unfavorable conditions during growth and reproductive processes, it makes perfect sense that healthy and strong plants have robust SI to favor outcrossing while weak or stressed plants would relax the control on SI to ensure some level of self-propagation. The fact that ethylene and PCD are often found associated with senescence and unfavorable conditions also enables plants to co-opt these pathways to balance SI and SC accordingly.

Our finding that ethylene-regulated PCD is associated with senescence-induced SI breakdown is an important advance for the horticultural breeding research and F1 hybrid seed production. Many of the Brassica family plants are widely cultivated and consumed all over the world; our study of Chinese cabbage and radish therefore has a broad range of applications. From the perspective of plant breeding, senescence-associated breakdown of SI reduces the purity of hybrid seeds and is better to be avoided or kept as low as possible. Understanding the mechanism of senescence-associated breakdown of SI might generate new approaches to eliminate self-fertilized seeds and to improve hybrid purity, such as boosting the overall health of the plants to delay the senescence process.

The breakdown of SI is equally important in the breeding industry because the propagation of SI inbred lines is the foundation for hybrid seed production. In practice, delayed pollination, i.e., pollination carried out on senescent stigmas, is utilized to overcome SI for the propagation of SI parental lines. Our finding that PCD is associated with senescence-induced SI breakdown explains the utilization of delayed pollination. Based on our finding that ethylene induces senescence-associated genes in stigmatic papilla cells (Figure 5 and Supplementary Figure 5; Su et al., 2020), we demonstrated that spraying 3–12 mM ethephon on the inflorescences of heading Chinese cabbage (Su et al., 2020), flowering Chinese cabbage (Figures 8A–F) and radish (Figures 8G–L), all significantly promoted the formation of self-fertilized seeds. Although the optimum concentration of ethephon need to be tested in different crops, ethephon spray has the potential to be implemented in the breakdown of SI in many cruciferous family crops (Figure 8 and Supplementary Figure 9).

## Conclusion

Our study shows that senescence of individual flowers and senescence of the plant both result in the breakdown of SI and the formation of self-fertilized seeds, affecting the purity of hybrid seeds in Chinese cabbage and in radish, and probably also other cruciferous family plants. Results show that age-dependent senescence upregulates ethylene, induces the expression of senescence-associated genes, and causes PCD in stigmatic papilla cells, which are closely associated with the breakdown of SI. Our findings are insightful to further reveal the mechanism underlying senescence-associated SI breakdown, also set the foundation for the prediction of SI under other unfavorable conditions, such as energy deprivation, drought, salinity, wounding, or various pathogen attacks. Knowledge produced here might help develop strategies to mitigate the impact of these unfavorable conditions on producing F1 hybrid. Our findings also led to the development of ethephon spray as a new strategy in overcoming SI for parental line propagation.

## Data Availability Statement

The raw data supporting the conclusions of this article will be made available by the authors, without undue reservation.

## Author Contributions

QD conceptualized and initiated the project. JH and SS contributed to all experiments with help from HD. CL and SW contributed to radish-related data. XW, YZ, ZW, XZ, and YY contributed to phenotypic observations. XY contributed to ethephon spray of flowering Chinese cabbage. CZ and YL contributed to transient gene silencing of Chinese cabbage flowers. QD, WZ, H-MW, and AC contributed to data analysis and presentation. QD wrote the manuscript. All authors participated in finalizing the manuscript. All authors contributed to the article and approved the submitted version.

## Conflict of Interest

The authors declare that the research was conducted in the absence of any commercial or financial relationships that could be construed as a potential conflict of interest.

## Footnotes

1 http://sfold.wadsworth.org/cgi-bin/soligo.pl

2 http://rna.tbi.univie.ac.at/cgi-bin/RNAWebSuite/RNAfold.cgi

3 https://blast.ncbi.nlm.nih.gov/Blast.cgi

## Supplementary Material

Supplementary Figure 1 Plant senescence leads to the formation of self-fertilized seeds in three varieties of Chinese cabbage. **(A)** Flowers at 0–6 day after anthesis (upper panel) and the corresponding stigmas (bottom panel) from old plants. Corresponding flowers and stigmas from young plants were shown in Figure 1A. **(B,C)** Flowers at 0-d from old plants were emasculated and pollinated at 0-4 day after emasculation (DAE) with self- or cross-pollen from young plants. **(C)** shows the number of pollen tubes penetrated each stigma. Corresponding stigmas from young plants were shown in Figure 1B. **(D)** Young and old plants of three varieties of Chinese cabbage. Chinese cabbage variety 14 CR is shown in Figure 2A. **(E)** Pistils at 0 DAE from young or old plants of three varieties of Chinese cabbage were pollinated with self-pollen. The growth and penetration of self-pollen tubes was observed at 6 HAP, to supplement data shown for 14 CR in Figure 2B. **(F)** Pistils at 0 DAE from young or old plants of three varieties of Chinese cabbage were pollinated with self-pollen. Pod length was measured at 12 DAP, to supplement data shown for 14 CR in Figure 2C. **(G)** Pistils at 0 DAE from young or old plants of three varieties of Chinese cabbage were pollinated with self-pollen. The number of seeds per pod was counted at 12 DAP, to supplement data shown for 14 CR in Figure 2D. Scale bars = 30 cm **(A)**; 500 μm **(B)**; 1 cm **(C,D)**. If not specified, ^∗^ and ^∗∗^, significant (*P* < 0.05) and highly significant difference (*P* < 0.01) between self- or cross-pollinated 0 DAE stigmas and their counterparts of 1–6 DAE stigmas, respectively. n.s. indicate no significant difference. ^∗^, ^∗∗^ or n.s. above the bracket show comparisons of these samples. *n* indicates the number of stigmas or pods tested.

Supplementary Figure 2 Senescence causes PCD in stigmatic papilla cells of Chinese cabbage. **(A)** The effect of self- or cross-pollination on papilla cell death, indicated by PI staining. Stigmas were unpollinated, or pollinated with self- or cross-pollen, then stained in PI at 60 min after pollination. Images of the upper panel were magnified from the outlined rectangular areas in the lower panel. **(B)** Whole image of papilla cell death in 0–6 DAE stigmas unpollinated or pollinated with self-pollen. Stigmas at 1 HAP were stained in PI. Images in Figure 3A were magnified from the outlined rectangular areas in **(C)**. **(C–E)** Stigmas at 0 DAE from young or old plants of three varieties of Chinese cabbage were unpollinated or pollinated with self-pollen. Stigmas at 1 HAP were stained in PI and observed for papilla cell death, to supplement data shown for 14 CR in Figure 3C. Images in **(D)** were magnified from the outlined rectangular areas in **(E)**. Scale bars = 100 μm (**A**, upper panel); 500 μm (**A**, lower panel); 500 μm **(C,E)**; 100 μm **(B,D)**. In **(A,C)**, arrows, PI stained cell wall of papilla cells; white stars, PI-stained nuclei of papilla cells; arrow heads, pollen grains. ^∗∗^, highly significant difference (*P* < 0.01). ^∗∗^ above the bracket show comparisons of these samples. n indicates the number of stigmas tested.

Supplementary Figure 3 Other hallmarks of PCD in senescent stigmas of Chinese cabbage. **(A)** Papilla cell death in 0 and 4 DAE stigmas, indicated by trypan blue staining. **(B,C)** FDA staining of 0–6 DAE stigmas. **(D–F)** Area of PI-stained nuclei and length/width ratio of PI-stained nuclei were demonstrated **(D)** and quantified **(E,F)**. Scale bars = 100 μm (**A**, upper panel; **B**, upper panel); 500 μm (**A**, lower panel; **B** lower panel). In **(A,B)**, arrow heads and white stars indicate live and dead cells, respectively. ^∗^ and ^∗∗^, significant (*P* < 0.05) and highly significant difference (*P* < 0.01). n.s. above the bracket show no significant difference of these samples. n indicates the number of stigmas tested.

Supplementary Figure 4 The expression of *SRK*_46_ in senescent Chinese cabbage stigmas. ^∗∗^, highly significant difference (*P* < 0.01). n.s. above the bracket show no significant difference of these samples.

Supplementary Figure 5 The expression of ethylene biosynthesis and response genes in senescent Chinese cabbage stigmas. **(A,B)** The expression of senescence-associated genes. *BrSAG12* (Br025046), *BrSAG29* (Br023394), and *BrORE1* (Br036223) were highly expressed and shown in Figures 4A,B. **(C)** Whole image of papilla cell death in stigmas mock treated or treated with S- or AS-BrSAG29. **(D)** Whole image of papilla cell death in stigmas mock treated or treated with S- or AS-BrORE1. ^∗∗^, highly significant difference.

Supplementary Figure 6 The expression of ethylene biosynthesis and response genes in senescent Chinese cabbage stigmas **(A,B).** The expression of *BrACS* genes. Among the *BrACS* genes tested, *BrACS6*, *BrACS9.2*, *BrACS10* and *BrACS12* were highly expressed and shown in Figures 5A,B. **(C)** The expression of *BrERF* genes. Among the *BrERF* genes tested, *BrERF109* (Bra034624), *BrERF107* (Bra016518), *BrERF012.1* (Bra012298) were highly expressed and shown in Figure 5D. ^∗∗^, highly significant difference.

Supplementary Figure 7 Senescent flowers and senescent radish plants. **(A)** Close up view of 0-d, 1-d, 2-d, 3-d, 4-d radish flowers and the corresponding stigmas (bottom panel). 0-d flower, just open with stigma lower than anthers and anthers not dehiscent; 1-d flower, 1 day after anthesis with stigma at the same level with anthers dehiscent; 2-d flower, 2 days after anthesis with stigmas longer than anthers and anthers wilt; 3-d flower, 3 days after anthesis with wilt anthers and flower start to close; 4-d flower, 4 days after anthesis that are completely closed. **(B)** Radish plants at young and old stages. Scale bars = 1 cm (**A**, upper panel); 100 μm (**A**, lower panel); 30 cm **(B)**.

Supplementary Figure 8 Papilla cell PCD in senescent radish stigmas. **(A)** Whole stigma image of papilla cell death in 0-4 DAE stigmas, indicated by PI staining. Images in Figure 7A were magnified from the outlined rectangular areas. **(B)** Whole stigma image of papilla cell death in stigmas of young or old radish plants, indicated by PI staining. Images in Figure 7C were magnified from the outlined rectangular areas. **(C)** FDA staining of 0–6 DAE stigmas. Scale bars = 500 μm **(A,B)**.

Supplementary Figure 9 Radish plants sprayed with ethephon produce viable self-fertilized seeds. **(A)** The image of 4-d-old seedlings from self-fertilized radish seeds after ethephon spray. **(B)** The germination rate of self-fertilized radish seeds after ethephon spray at 4 day after germination. Scale bar = 1 cm **(A)**. n.s, no significant difference. *n* indicates the number of seeds tested.

Supplementary Table 1 Primers used in this study.

Supplementary Table 2 S or AS-ODN Sequence.
