# Supplementary figures and images for: Programmed Cell Death in Stigmatic Papilla Cells Is Associated With Senescence-Induced Self-Incompatibility Breakdown in Chinese Cabbage and Radish
Source: Front Plant Sci. 2020 Dec 7;11:586901. doi: 10.3389/fpls.2020.586901 (PMC7750362; doi:10.3389/fpls.2020.586901)

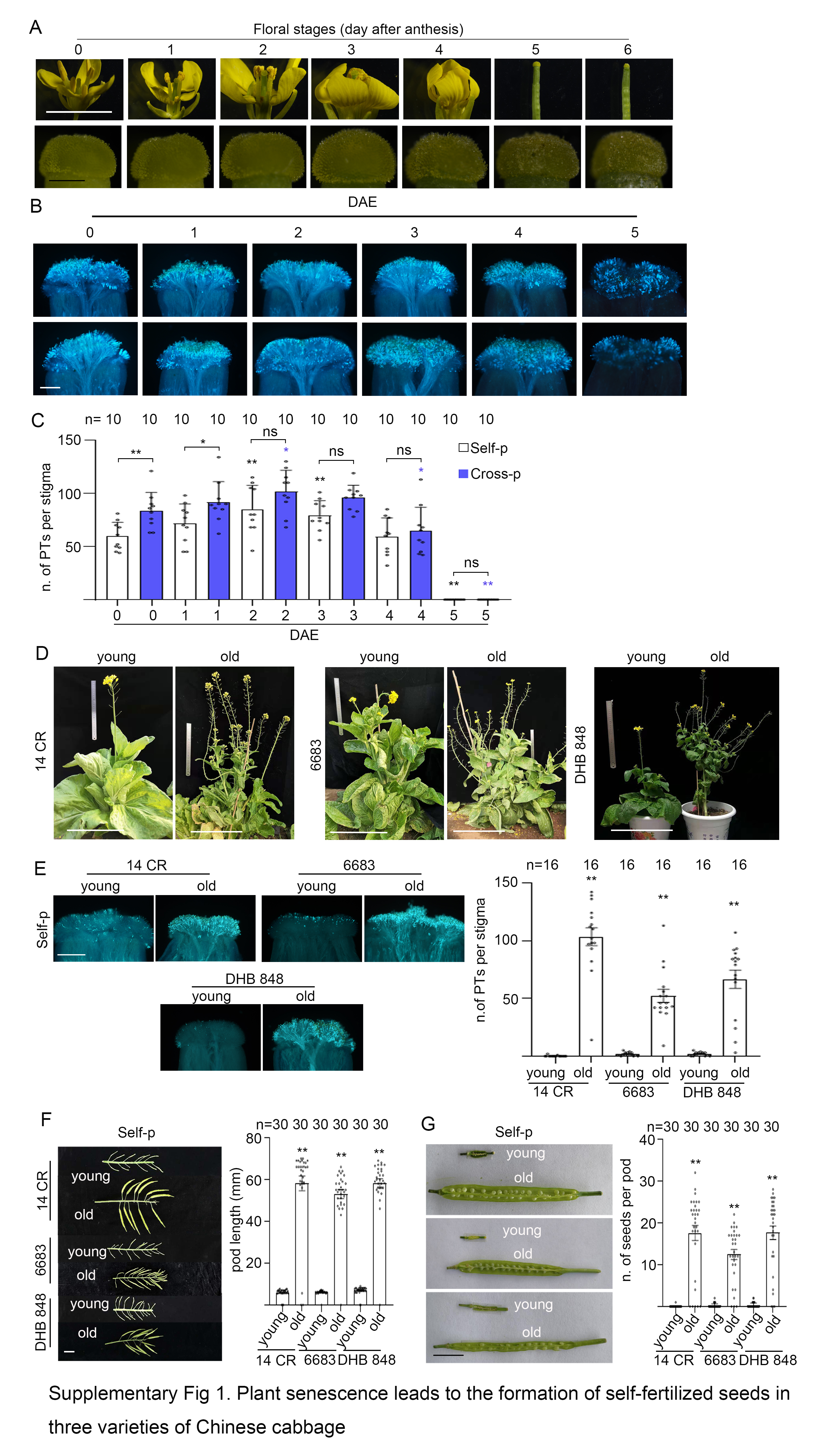

Supplement: Supplementary Figure 1 — Plant senescence leads to the formation of self-fertilized seeds in three varieties of Chinese cabbage. (A) Flowers at 0–6 day after anthesis (upper panel) and the corresponding stigmas (bottom panel) from old plants. Corresponding flowers and stigmas from young plants were shown in Figure 1A. (B,C) Flowers at 0-d from old plants were emasculated and pollinated at 0-4 day after emasculation (DAE) with self- or cross-pollen from young plants. (C) shows the number of pollen tubes penetrated each stigma. Corresponding stigmas from young plants were shown in Figure 1B. (D) Young and old plants of three varieties of Chinese cabbage. Chinese cabbage variety 14 CR is shown in Figure 2A. (E) Pistils at 0 DAE from young or old plants of three varieties of Chinese cabbage were pollinated with self-pollen. The growth and penetration of self-pollen tubes was observed at 6 HAP, to supplement data shown for 14 CR in Figure 2B. (F) Pistils at 0 DAE from young or old plants of three varieties of Chinese cabbage were pollinated with self-pollen. Pod length was measured at 12 DAP, to supplement data shown for 14 CR in Figure 2C. (G) Pistils at 0 DAE from young or old plants of three varieties of Chinese cabbage were pollinated with self-pollen. The number of seeds per pod was counted at 12 DAP, to supplement data shown for 14 CR in Figure 2D. Scale bars = 30 cm (A); 500 μm (B); 1 cm (C,D). If not specified, ∗ and ∗∗, significant (P < 0.05) and highly significant difference (P < 0.01) between self- or cross-pollinated 0 DAE stigmas and their counterparts of 1–6 DAE stigmas, respectively. n.s. indicate no significant difference. ∗, ∗∗ or n.s. above the bracket show comparisons of these samples. n indicates the number of stigmas or pods tested. [file Image_1.JPEG]

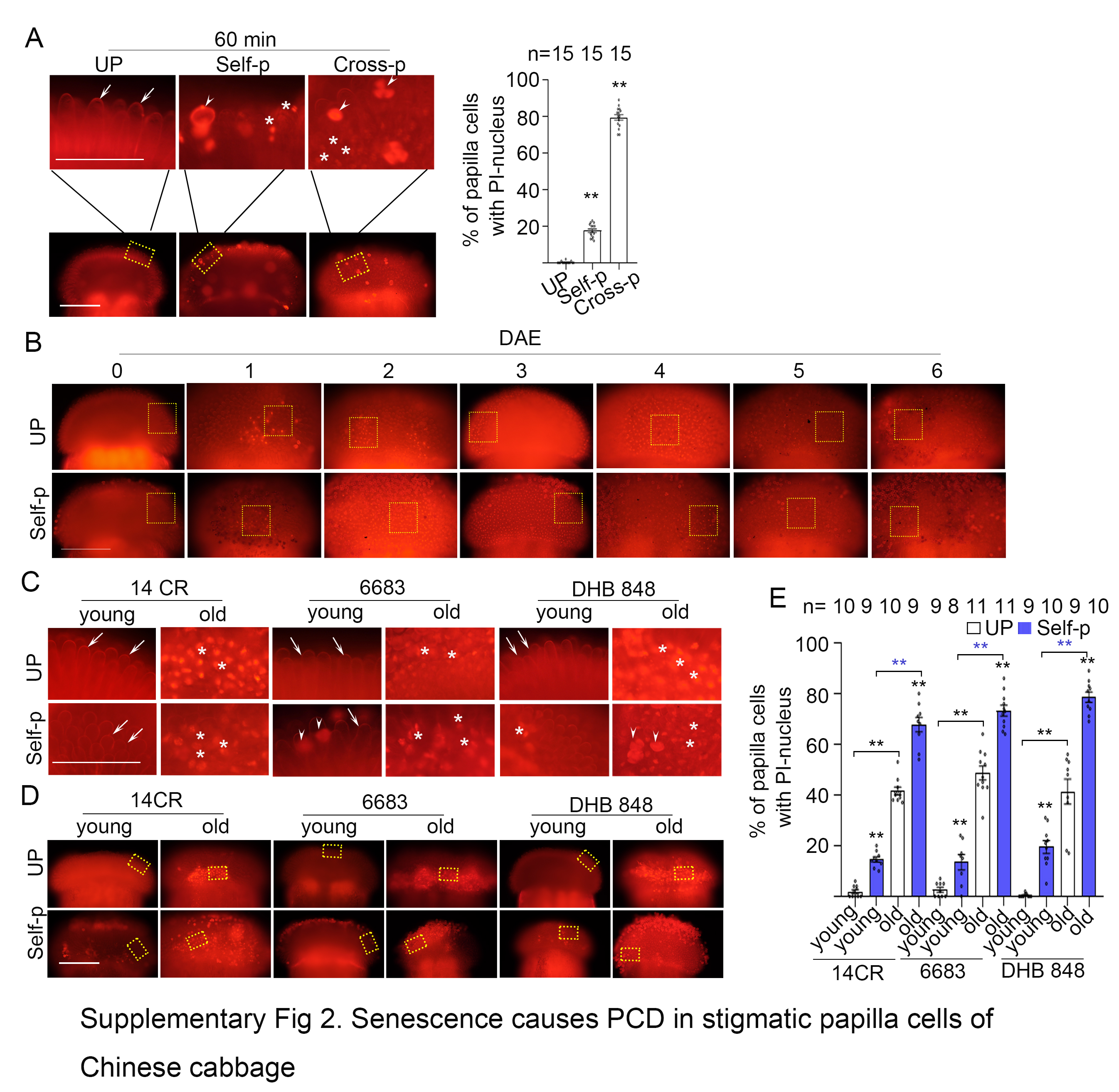

Supplement: Supplementary Figure 2 — Senescence causes PCD in stigmatic papilla cells of Chinese cabbage. (A) The effect of self- or cross-pollination on papilla cell death, indicated by PI staining. Stigmas were unpollinated, or pollinated with self- or cross-pollen, then stained in PI at 60 min after pollination. Images of the upper panel were magnified from the outlined rectangular areas in the lower panel. (B) Whole image of papilla cell death in 0–6 DAE stigmas unpollinated or pollinated with self-pollen. Stigmas at 1 HAP were stained in PI. Images in Figure 3A were magnified from the outlined rectangular areas in (C). (C–E) Stigmas at 0 DAE from young or old plants of three varieties of Chinese cabbage were unpollinated or pollinated with self-pollen. Stigmas at 1 HAP were stained in PI and observed for papilla cell death, to supplement data shown for 14 CR in Figure 3C. Images in (D) were magnified from the outlined rectangular areas in (E). Scale bars = 100 μm (A, upper panel); 500 μm (A, lower panel); 500 μm (C,E); 100 μm (B,D). In (A,C), arrows, PI stained cell wall of papilla cells; white stars, PI-stained nuclei of papilla cells; arrow heads, pollen grains. ∗∗, highly significant difference (P < 0.01). ∗∗ above the bracket show comparisons of these samples. n indicates the number of stigmas tested. [file Image_2.JPEG]

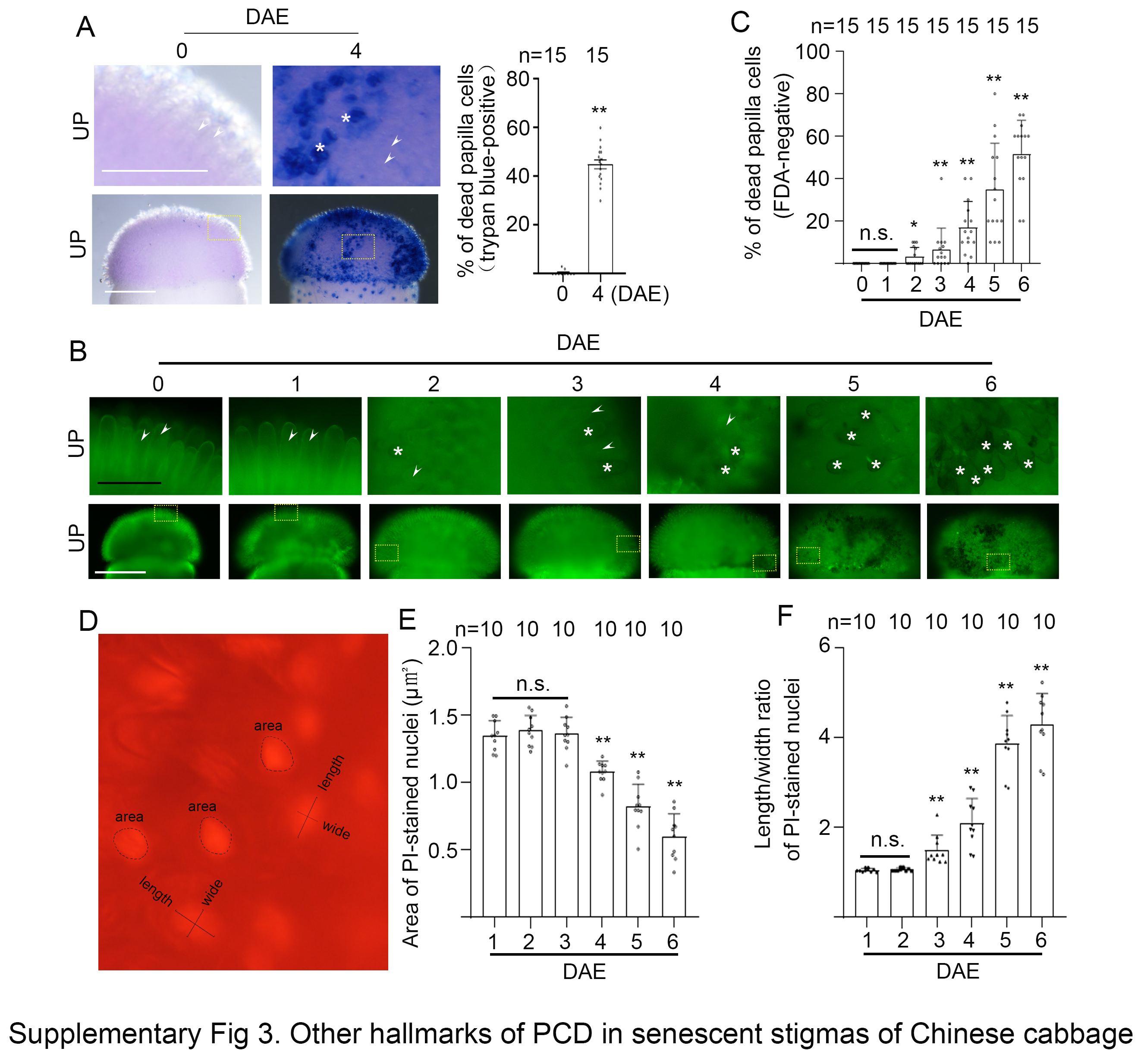

Supplement: Supplementary Figure 3 — Other hallmarks of PCD in senescent stigmas of Chinese cabbage. (A) Papilla cell death in 0 and 4 DAE stigmas, indicated by trypan blue staining. (B,C) FDA staining of 0–6 DAE stigmas. (D–F) Area of PI-stained nuclei and length/width ratio of PI-stained nuclei were demonstrated (D) and quantified (E,F). Scale bars = 100 μm (A, upper panel; B, upper panel); 500 μm (A, lower panel; B lower panel). In (A,B), arrow heads and white stars indicate live and dead cells, respectively. ∗ and ∗∗, significant (P < 0.05) and highly significant difference (P < 0.01). n.s. above the bracket show no significant difference of these samples. n indicates the number of stigmas tested. [file Image_3.JPEG]

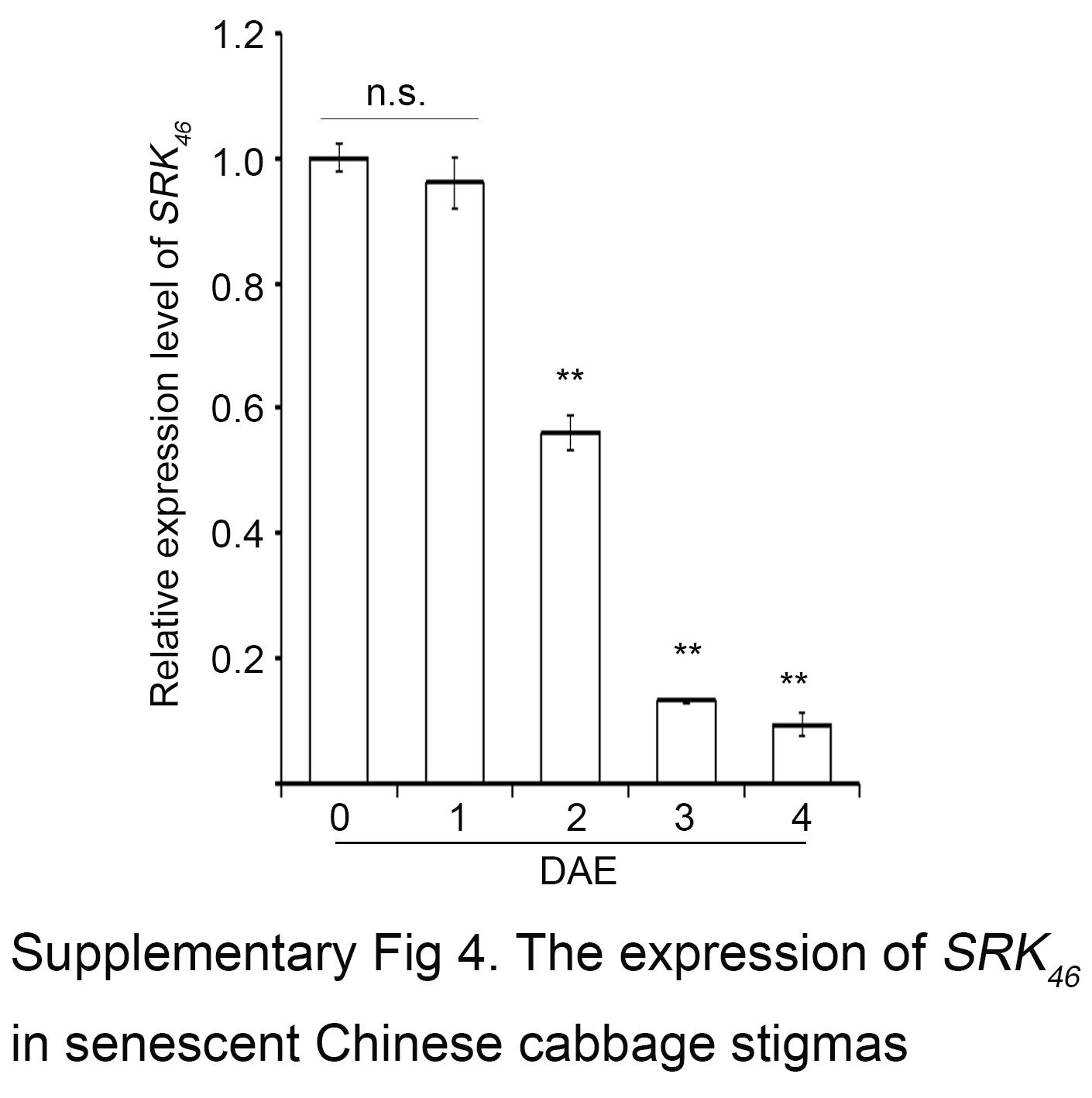

Supplement: Supplementary Figure 4 — The expression of SRK46 in senescent Chinese cabbage stigmas. ∗∗, highly significant difference (P < 0.01). n.s. above the bracket show no significant difference of these samples. [file Image_4.JPEG]

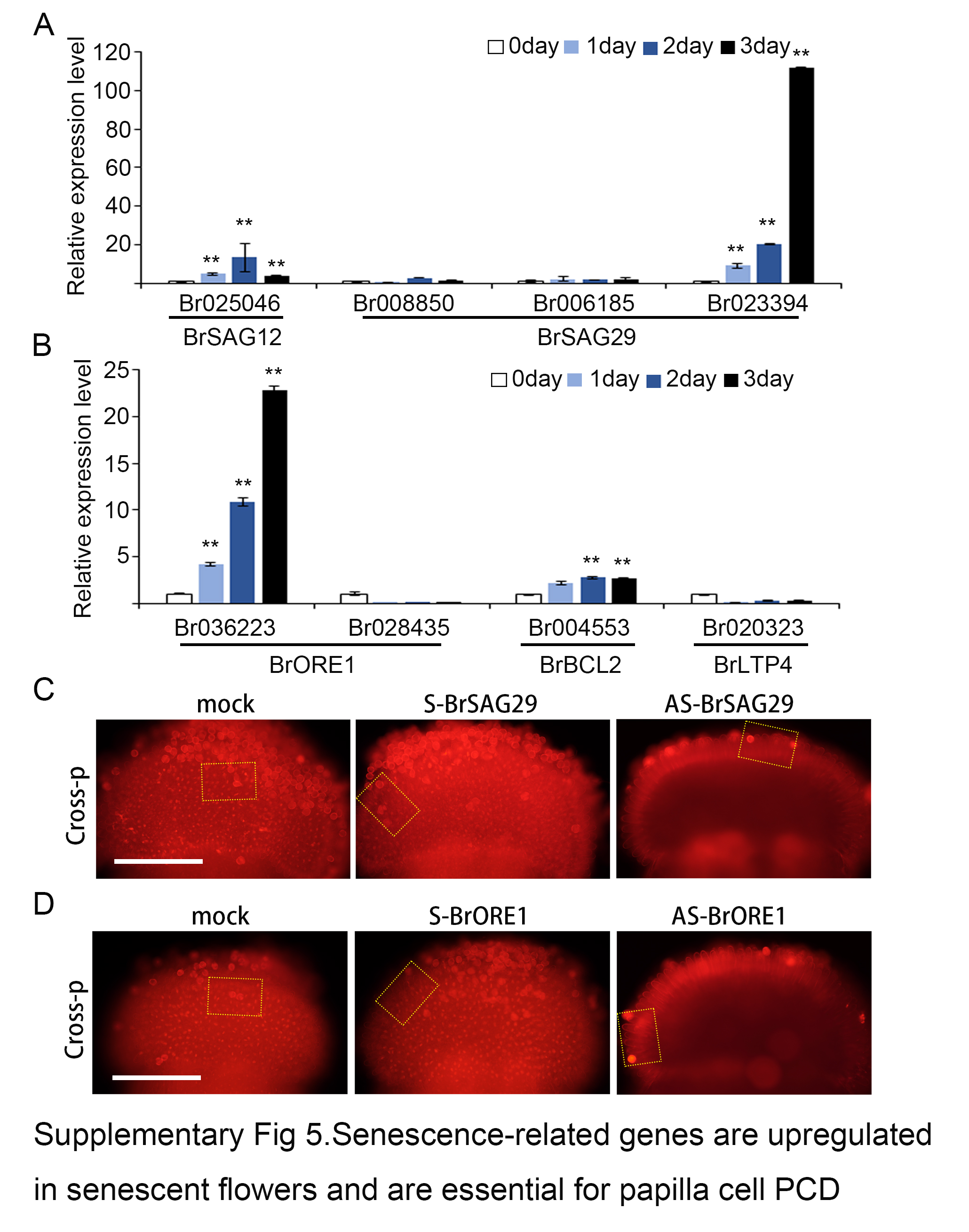

Supplement: Supplementary Figure 5 — The expression of ethylene biosynthesis and response genes in senescent Chinese cabbage stigmas. (A,B) The expression of senescence-associated genes. BrSAG12 (Br025046), BrSAG29 (Br023394), and BrORE1 (Br036223) were highly expressed and shown in Figures 4A,B. (C) Whole image of papilla cell death in stigmas mock treated or treated with S- or AS-BrSAG29. (D) Whole image of papilla cell death in stigmas mock treated or treated with S- or AS-BrORE1. ∗∗, highly significant difference. [file Image_5.JPEG]

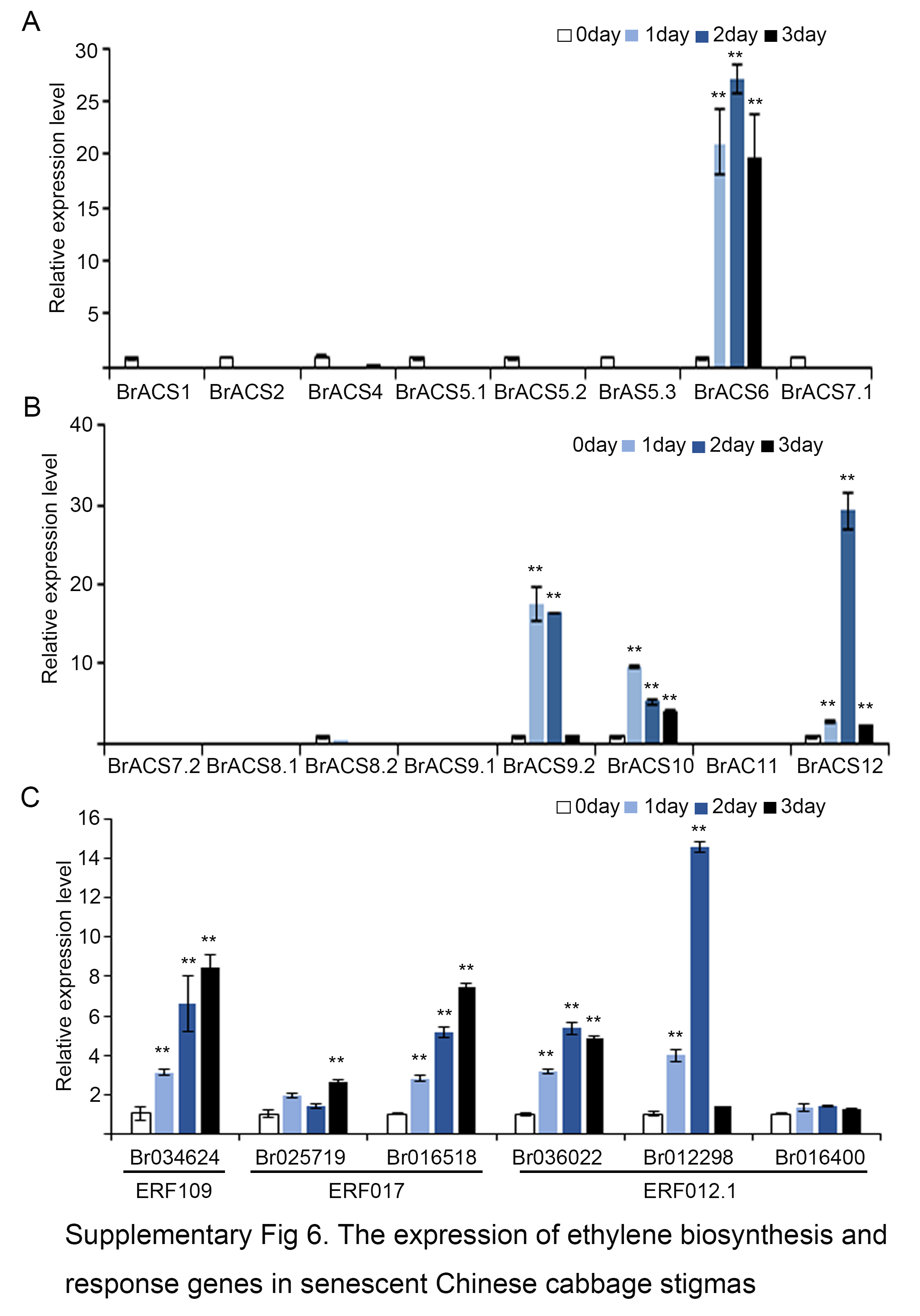

Supplement: Supplementary Figure 6 — The expression of ethylene biosynthesis and response genes in senescent Chinese cabbage stigmas (A,B). The expression of BrACS genes. Among the BrACS genes tested, BrACS6, BrACS9.2, BrACS10 and BrACS12 were highly expressed and shown in Figures 5A,B. (C) The expression of BrERF genes. Among the BrERF genes tested, BrERF109 (Bra034624), BrERF107 (Bra016518), BrERF012.1 (Bra012298) were highly expressed and shown in Figure 5D. ∗∗, highly significant difference. [file Image_6.JPEG]

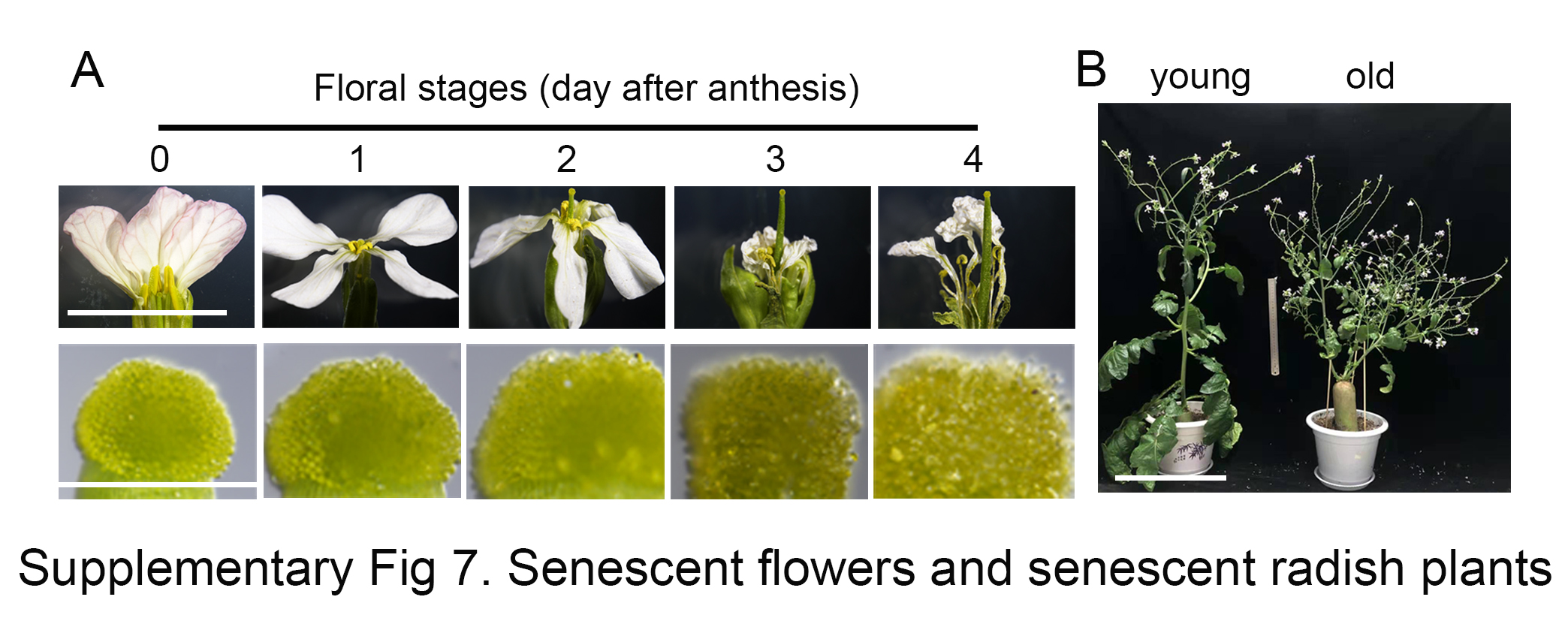

Supplement: Supplementary Figure 7 — Senescent flowers and senescent radish plants. (A) Close up view of 0-d, 1-d, 2-d, 3-d, 4-d radish flowers and the corresponding stigmas (bottom panel). 0-d flower, just open with stigma lower than anthers and anthers not dehiscent; 1-d flower, 1 day after anthesis with stigma at the same level with anthers dehiscent; 2-d flower, 2 days after anthesis with stigmas longer than anthers and anthers wilt; 3-d flower, 3 days after anthesis with wilt anthers and flower start to close; 4-d flower, 4 days after anthesis that are completely closed. (B) Radish plants at young and old stages. Scale bars = 1 cm (A, upper panel); 100 μm (A, lower panel); 30 cm (B). [file Image_7.JPEG]

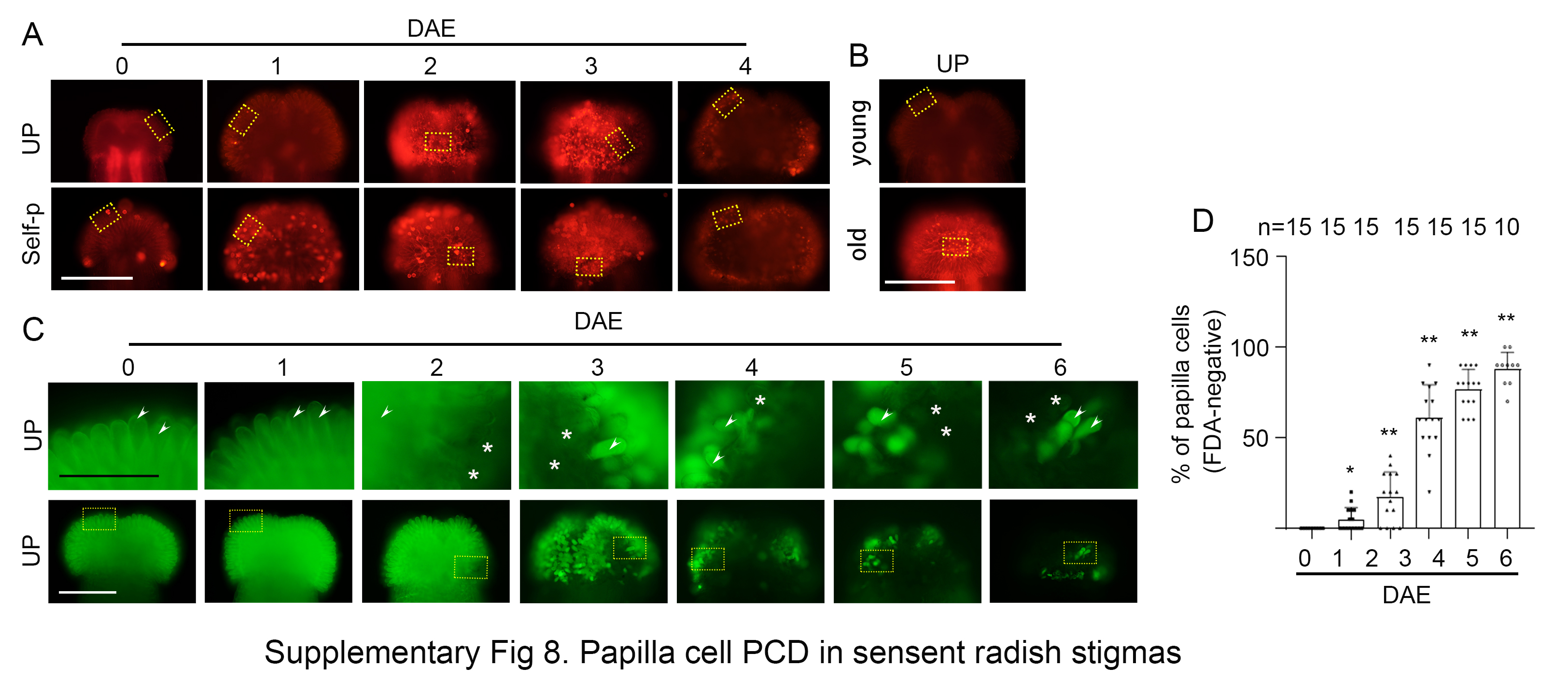

Supplement: Supplementary Figure 8 — Papilla cell PCD in senescent radish stigmas. (A) Whole stigma image of papilla cell death in 0-4 DAE stigmas, indicated by PI staining. Images in Figure 7A were magnified from the outlined rectangular areas. (B) Whole stigma image of papilla cell death in stigmas of young or old radish plants, indicated by PI staining. Images in Figure 7C were magnified from the outlined rectangular areas. (C) FDA staining of 0–6 DAE stigmas. Scale bars = 500 μm (A,B). [file Image_8.JPEG]

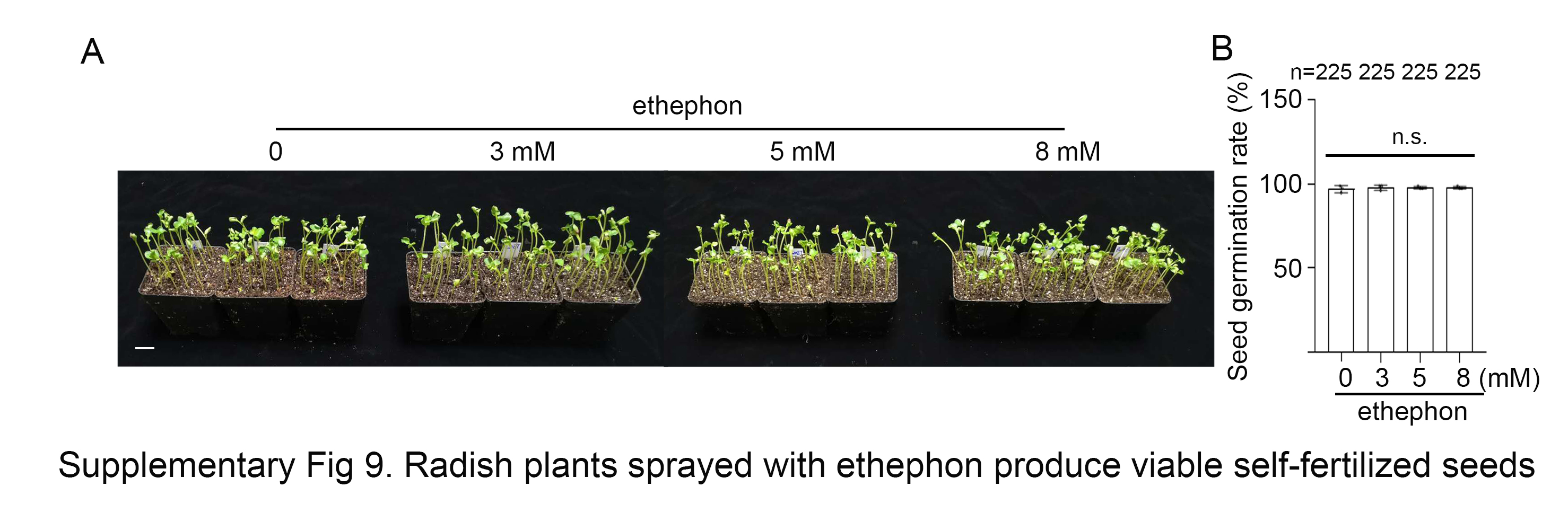

Supplement: Supplementary Figure 9 — Radish plants sprayed with ethephon produce viable self-fertilized seeds. (A) The image of 4-d-old seedlings from self-fertilized radish seeds after ethephon spray. (B) The germination rate of self-fertilized radish seeds after ethephon spray at 4 day after germination. Scale bar = 1 cm (A). n.s, no significant difference. n indicates the number of seeds tested. [file Image_9.JPEG]
