# Supplementary material for: Programmed Cell Death in Stigmatic Papilla Cells Is Associated With Senescence-Induced Self-Incompatibility Breakdown in Chinese Cabbage and Radish
Source: Front Plant Sci. 2020 Dec 7;11:586901. doi: 10.3389/fpls.2020.586901 (PMC7750362; doi:10.3389/fpls.2020.586901)
Supplement: Supplementary Table 1 — Primers used in this study. [file Data_Sheet_1.PDF]

Supplementary Table 1. The primers in this study

Primer		Sequence5'-3'	
BrActin-2	qBrActin-2-F	CGGTGTCATGGTTGGGATGA	
	qBrActin-2-R	CGTGCTCGATGGGGTACTTC	
BrSRK46	qBrSRK46-F	AAAAGGAGGACACGGCTGAG	
	qBrSRK46-R	TTCACACCCATACTGCGGTC	
BrSAG12	qBr025046-F	GGTTTGTCGGAAATGCACCC	
	qBr025046-R	TTTGTGCGGCTTCAGTTGAC	
BrSAG-29	qBr008850-F	GGACTGAACCAGTGGACCC	
	qBr008850-R	TTCATGACGGGCAGTTTCA	
	qBr006185-F	GCACACCCCAATGATGAAGC	
	qBr006185-R	GCGACATTGGTACGGTAGT	
	qBr023394-F	GGTTTGTCGGAAATGCACCC	
	qBr023394-R	TTTGTGCGGCTTCAGTTGAC	
BrORE1	qBr036223-F	CGGTTTACCTCCCCTGATGG	
	qBr036223-R	CTCGGTTGTTTGGTCGGAGA	
	qBr028435-F	ACCGAACTGAACCGACAAGA	
	qBr028435-R	CGACGAACCACGGAAGGAAT	
BrBCL2	qBr004553-F	GAAGCCTGCGAAGAAGACCT	
	qBr004553-R	GCGCCTGAACACGCTTTTTA	
BrLTP4	qBr020323-F	ATCCAAGTCTAGCCTCCGGC	
	qBr020323-R	TTGTCGCAGTTGGTGCTCAT	qRT-PCR
BrACS1	qBr007611-F	ACATGGGTCTTCCCGGTTTC	
	qBr007611-R	CTTGCACAGGTCACAACAGC	
BrACS2	qBr033261-F	CGATCCCGGCGATGTTTTTC	
	qBr033261-R	ACGGCCTCGACGGTTAATTT	
BrACS4	qBr030199-F	AAAGAATGCTCGTGACGGGT	
	qBr030199-R	TCTTCACAATGGCACGACGA	
BrACS5.1	qBr031846-F	GGGAGCGATCTACTCCAACG	
	qBr031846-R	GACCAGACACTAGGCGCTTT	
BrACS5.2	qBr024420-F	AACGGCTCAAGTCCAGACAG	
	qBr024420-R	CAACAGAACAAACCGGCGTT	
BrACS5.3	qBr037829-F	GGGAGCGATCTACTCCAACG	
	qBr037829-R	GTCCAAGAGGTGTCTGAGCC	
BrACS6	qBr035236-F	AGATCACGGTGGAAGCCTTG	
	qBr035236-R	CGGTCTAACGTCGTACCGAG	
BrACS7.1	qBr019117-F	ACATCGTTTACAGCCTCTCCA	
	qBr019117-R	TGTGTTGTGTCTGGGACGAG	
BrACS7.2	qBr026456-F	AGGCAGGGATCGAGTGTTTG	
	qBr026456-R	GCACGAAGATCCAGGCGATA	
BrACS8.1	qBr010611-F	TGGCGGATTTCATGTCGGAA	

	qBr010611-R	TCCAGGATCAGCGAGACAGA	
BrACS8.2	qBr011784-F	ACGCTTTCTTGCTGCCTACT	
	qBr011784-R	CGATCTCAGCTCCGGTTCTC	
BrACS9.1	qBr017962-F	GAGAACCGGAGCAGAGATCG	
	qBr017962-R	TCTGGTCAACATCGTGCCAA	
BrACS9.2	qBr012924-F	CTCTCTGCACTGCTTTCCGA	
	qBr012924-R	TCAAGACCGGACACGAGTTG	
BrACS10	qBr027008-F	AATCCGCGGCGTCATAATCT	
	qBr027008-R	TGGACAGAACCAGCGAAGAC	
BrACS11	qBr000637-F	CGACCTGATAGAGTCGTGGC	
	qBr000637-R	GCATCTTTGAAAGCCGGCAA	
BrACS12	qBr022560-F	CTTCGTCGGCTCTCTCATCC	
	qBr022560-R	CTCCGTCGAGTCGGAATCAG	
BrCTR1	qBrCTR1-F	GGCATATGATGTGGTTTGTGAT	
	qBrCTR1-R	TTCCAGCTGCCGACTTTGAT	
BrERF109	qBr034624-F	TTTCGAACCCGCTGAGGATG	qRT-PCR
	qBr034624-R	AGTGTCAGCTGCAACAGGAG	
BrERF017	qBr025719-F	AGGAGATGGGGGAAATGGGT	
	qBr025719-R	AAATTGAAATTCGCGCCGCT	
	qBr016518-F	GGCGCCAAGTTCAATTTCCC	
	qBr016518-R	CGCCACACTCTCTTCCGAAT	
BrERF012.1	qBr036022-F	ATCCATCCAAAGAGTCGCCG	
	qBr036022-R	CGGTGACGAGGTAGCAGATG	
	qBr012298-F	ATTTCGTCAGCCGTCTCGTC	
	qBr012298-R	AAGACACACGACAGTCCACG	
	qBr016400-F	AACTTCCCCAGCTCTGCTTC	
	qBr016400-R	ATCATGATCGGACGGCGAC	
