# Supplementary material for: Programmed Cell Death in Stigmatic Papilla Cells Is Associated With Senescence-Induced Self-Incompatibility Breakdown in Chinese Cabbage and Radish
Source: Front Plant Sci. 2020 Dec 7;11:586901. doi: 10.3389/fpls.2020.586901 (PMC7750362; doi:10.3389/fpls.2020.586901)
Supplement: Supplementary Table 2 — S or AS-ODN Sequence. [file Data_Sheet_2.PDF]

Supplementary Table 2. S or AS-ODN Sequence

S-BrSAG29	GCGGCCATGAAGTTGTTCTT
AS-BrSAG29	AAGAACAACTTCATGGCCGC
S- BrORE1	GCCATTGGTGAAGTTGATCTC
AS- BrORE1	GAGATCAACTTCACCAATGGC
